# Extraction and Optimization of Fucoidan From *Sargassum muticum* Using Acidic Eutectic Solvents and Evaluation of Their Effects on Fibroblast Cell Lines

**DOI:** 10.1002/open.202500448

**Published:** 2026-04-01

**Authors:** Marina Leite, Francisca Silva, Antônio Lima, Afonso Cunha, Thiago Cahú, Helga Gomes, Paulo A. S. Mourão, Mauro S. G. Pavão, Bernardo D. Ribeiro

**Affiliations:** ^1^ Department of Biochemical Enginnering School of Chemistry Federal University of Rio de Janeiro Rio de Janeiro Brazil; ^2^ Institution of Medical Biochemistry Leopoldo de Meis Federal University of Rio de Janeiro Rio de Janeiro Brazil

**Keywords:** cellular regeneration, deep eutectic solvents, fucoidan, healing, *Sargassum muticum*

## Abstract

Fucoidan, a sulfated polysaccharide extracted from the cell wall of macroalgae such as *S. muticum*, exhibits promising biological properties. This study investigated a green extraction using deep eutectic solvents (DESs), with an emphasis on process optimization and bioactive evaluation of fucoidan. The most efficient DES was lactic acid with glycerol (LacAc:Gly) at 60°C, with 4% water and a solid/liquid ratio of 0.1167, resulting in a yield of 6.6%, a sulfate content of 0.170 mg/mg, and a uronic acid content of 0.023 mg/mg. The chemical and structural composition of fucoidan were evaluated by colorimetric methods, FTIR, and NMR. Antioxidant activity was assessed by 2,2′‐azino‐bis(3‐ethylbenzothiazoline‐6‐sulfonate (ABTS, 25.84%), 2‐diphenyl‐1‐pricylhydrazyl (DPPH, 30.45%), and ferric reducing antioxidant power (FRAP, 690 µg/µg) assays. Cellular assays with L929 fibroblasts showed that concentrations of 50 and 75 µg/mL stimulated cell viability, with 179% colony formation and 449 invasive cells, suggesting positive effects on cell proliferation and migration. Therefore, the results indicate that fucoidan extracted with LacAc:Gly has high bioactive potential, demonstrating the viability of green eutectic solvents as a sustainable alternative for the utilization of bioactive compounds from brown algae.

## Graphical abstract

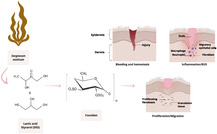

## 1 Introduction

Marine macroalgae are abundant sources of a variety of bioactive compounds in their chemical composition, including polysaccharides, minerals, vitamins, polyphenols, pigments, hormones, and lipids [1]. In this context, *Sargassum muticum* stands out, a brown macroalgae described for its rapid growth, high abundance, and diversity of bioactive metabolites, characteristics that make it a sustainable and promising alternative for various industrial and biotechnological applications [2, 3, 4, 5].

Polysaccharides are essential components of seaweed extracts, accounting for up to 30%–40% of the dry mass of these organisms, which present complex structural characteristics, with several monosaccharide units, types of bonds, degrees of branching, and varied molecular weights [6, 7]. In particular, fucoidan, a sulfated polysaccharide present in the cell wall of brown macroalgae, has a complex and heterogeneous chemical structure, characterized by the predominant presence of L‐fucose in its composition and smaller proportions of galactose, xylose, rhamnose, glucose, mannose, arabinose, and glucuronic acid linked by *α*−1,3 and *α*−1,4 glycosidic bonds [8].

Traditional methods for fucoidan extraction include acid, alkaline, and enzymatic treatment [9]. However, considering the economic and environmental aspects involved in the extraction of polysaccharides from *S. muticum*, the use of conventional solvents has limitations due to their high toxicity, low biodegradability, and difficult recycling [9, 10] Alternatively, deep eutectic solvents (DESs) are used, formed by the mixture of a hydrogen bond donor (HBD) and an acceptor (HBA), resulting in a solvent with a lower melting point than the individual components [9, 11].

Fucoidans extracted from *S. muticum* using traditional methods have demonstrated anti‐inflammatory properties, with the ability to interact with signaling molecules and antioxidants, such as transforming growth factor *β*1 (TGF‐*β*1), superoxide dismutase (SOD), glutathione (GSH), and malondialdehyde (MDA), and are relevant in studies of tissue healing and regeneration [12, 13]. Some studies have also reported the incorporation of fucoidans in alginate‐based dressings, showing positive effects on wound repair and accelerated cell growth [14, 15].

The aim of this study was to develop and optimize an innovative method for the extraction of fucoidan from the brown seaweed *S. muticum*, using new acidic eutectic solvents, and to evaluate the antioxidant and inflammatory potential of L929 fibroblast cell lines.

## 2 Results and Discussion

### 2.1 Chemical Composition of *S. muticum* Biomass

**FIGURE 1:**
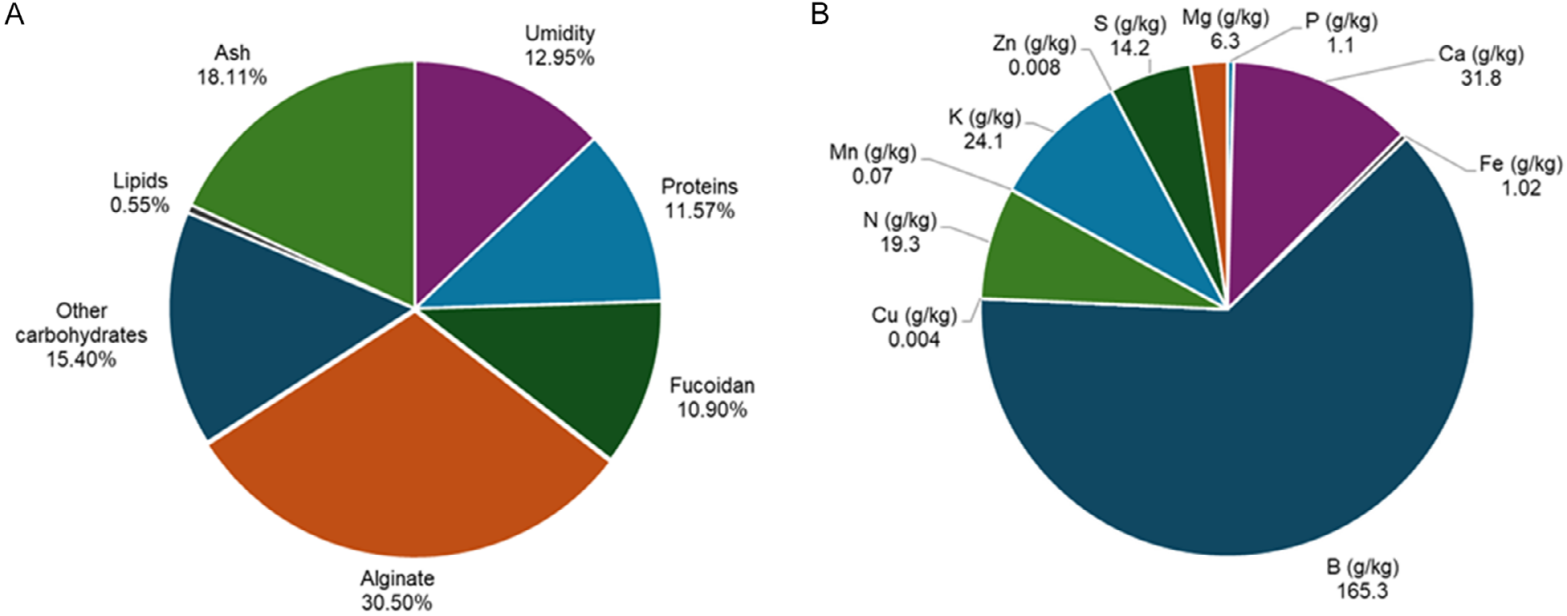
Composition of *S. muticum*: (A) Chemical composition. (B) Mineral composition.

The brown macroalgae *S. muticum* is composed of different groups of macromolecules, with promising biotechnological potential. The analysis of these constituents is shown in Figure 1A. The compositional data presented were obtained from dried *S. muticum* biomass. Fucoidan and alginate were isolated using conventional extraction methods to evaluate the carbohydrate and structural polysaccharide content of the species. The results obtained showed that *S. muticum* has a typical composition of brown algae, characterized by high levels of structural polysaccharides. The alginate content (30.5%) was within the range described for this species, which varied between 5.1% and 34.6%. Mohamed et al. [16] reported an alginate content of 24.66%, which represents the largest structural fraction of the *S. muticum* cell wall. In coastal environments, the intensity of waves and currents demands structural resistance, which is consistent with the high alginate content, acting as an adaptive component of macroalgae [17, 18]. The fucoidan content (10.9%) is consistent for brown algae. In the study by González‐Ballesteros [19], yields between 9.8% and 10.4% were observed, indicating that the value obtained is within the expected range, reinforcing the species’ potential for industrial applications. From a biological perspective, fucoidan plays a fundamental structural and protective role in the cell wall of algae, acting as a barrier against pathogens and microorganisms, as well as contributing to protection against abiotic stresses, such as UV radiation and salinity variations [20, 21].

The protein fraction (11.57%) indicated the presence of glycoprotein. As reported by Mouga et al. [22], they present approximately 10.25% protein, that is, a value corresponding to the algae matrix, which is part of the structural and metabolic functions of the macroalgae, in addition to assisting in the integrity and organization of the cellular structure. The ash content (18.11%) represents a high mineral load, which may be advantageous for nutraceutical use. However, for pharmaceutical processes, a purification step will be necessary, as it requires greater purity [23]. Studies such as Mouga et al. reported approximately 28.42% ash, a higher content than that of the study in question [22]. The lipid content (0.55%) presented low levels and may contain fractions of polyunsaturated fatty acids or lipophilic antioxidant compounds [22, 24, 25]. The residual moisture content of 12.95% indicates a safe range for storing the seaweed, without microbial degradation, being a fundamental condition for preserving the stability of the bioactive compounds [22, 26, 27].

Analysis of the nutritional composition of the *S. muticum* matrix indicates not only its environmental adaptation but also its relevance as a renewable resource for various industries. The mineral composition of *S. muticum* (Figure 1B) showed significant concentrations of macro and micronutrients, indicating that it is a biomass rich in essential elements. The high levels of calcium (31.8 g/kg), potassium (24.1 g/kg) and boron (165,3 g/kg) indicate greater adaptation of this macroalgae to the environment, as these minerals are responsible for the structure of the cell wall [28, 29]. Calcium is important in the formation of alginate gels, making them firmer, which is explained by the high alginate content (30.5%) found in the biomass. Therefore, it can be indicated that the cell walls are thick and resistant to external variations [28].

The considerably high sulfur content (14.2 g/kg) is attributed to sulfated compounds such as fucoidan, indicating the importance of this polysaccharide in the algae's defense [29]. Furthermore, the macroalgae presented an iron content of 1.02 g/kg, representing a natural source of this component, indicating high metabolic activities, since they are responsible for the transport of electrons in photosynthesis [29, 30, 31]. Thus, the mineral composition present in *S. muticum* is related to the structural and metabolic elements, in addition to the potential (nutritional and functional formulations) of the macroalgae.

### 2.2 Characterization of DESs

The DES formulation used in this study was based on low pH and high polarity systems, capable of selectively extracting fucoidan from the algae *S. muticum*, without extracting other polysaccharides such as alginate. In this context, the combination of organic acids and polyols was chosen instead of using quaternary salts such as choline chloride. This choice was based on studies that demonstrate that systems composed of organic acids and polyols have lower toxicity and are equally efficient in interacting with the algal matrix [32, 33, 34].

From a structural point of view, organic acids contribute to an acidic pH (increasing the solubilization of sulfated polysaccharides), while polyols (rich in hydroxyl groups) aid in the dense hydrogen bonding network, resulting in greater structural stability of the DESs [32, 33]. Based on these principles, this study analyzed 24 eutectic solvents with different physicochemical properties (Table 1). It was observed that glycerol‐based eutectics with lactic acid, acetic acid, and propionic acid exhibited high viscosity. Similar results were reported by Liu et al. [35], who reported viscosity values of 350 mPa.s for glycerol eutectics, which may be related to hydrogen bonding and polyol structures.

**TABLE 1:** Eutectic solvents used in the study and their respective properties.

Eutectic solvent	Abbreviation	Molar ratio	pH	Td, °C
1,2‐Propanediol:Acetic acid	12PDO:AcOH	1.20	0.83	111
1,2‐Propanediol:Lactic acid	12PDO:LacAc	1.80	1.36	139
1,2‐Propanediol:Propionic acid	12PDO:PropAc	1.40	1.92	107
Acetic acid:Glycerol	AcOH:Gly	1.70	1.55	61
Acetic acid:Trimethylolpropane	AcOH:TMP	2.10	0.40	63
Lactic acid:Glycerol	LacAc:Gly	1.20	0.87	218
Lactic acid:Proline	LacAc:Pro	1.90	2.71	218
Lactic acid:Trimethylolpropane	LacAc:TMP	1.50	0.91	229
Propionic acid:Glycerol	PropAc:Gly	1.80	0.54	69
Propionic acid:Trimethylolpropane	PropAc:TMP	2.10	0.90	76
1,2‐Propanediol:Glycolic acid	12PDO:GlycAc	3.10	1.51	128
Glycerol:Glycolic acid	Gly:GlycAc	1.30	0.89	217
1,3‐Butanediol:Glycolic acid	13BDO:GlycAc	2.10	1.80	188
1,3‐Butanediol:Lactic acid	13BDO:LacAc	1.40	1.54	170
Propionic acid:1,3‐Butanediol	PropAc:13BDO	1.10	1.38	217
Trimethylolpropane:Glycolic acid	TMP:GlycAc	1.10	1.45	253
1,5‐Pentanediol:Glycolic acid	15PDO:GlycAc	1.90	2.73	217
Acetic acid:1,3‐Butanediol	AcOH:13BDO	1.10	1.93	133
Acetic acid:1,5‐Pentanediol	AcOH:15PDO	1.70	1.70	150
Lactic acid:1,5‐Pentanediol	LacAc:15PDO	1.10	1.97	187
1,4‐Butanediol:Glycolic acid	14BDO:GlycAc	1.50	2.00	50
Acetic acid:1,4‐Butanediol	AcOH:14BDO	1.50	1.32	140
Lactic acid:1,4‐Butanediol	LacAc:14BDO	1.00	1.93	184
Propionic acid:1,4‐Butanediol	PropAc:14BDO	1.50	1.82	126

The DES formulation was based on a low pH and high polarity system, capable of promoting the selective extraction of fucoidan from seaweed biomass without coextraction of alginate. Therefore, simulations with COSMO‐RS (Table SA1) were employed as a screening tool to evaluate the relative affinities between fucoidan and the solvent and to identify favorable eutectic systems. The simulations indicated that all eutectic solvents presented low activity coefficients for fucoidan (represented by the 1314 octamer of fucoidan), suggesting high solubility. This behavior is probably associated with the acidic conditions of the systems, in which the pH values were below the pKa of fucoidan (Table 1). Among the systems evaluated, the best performances predicted by COSMO‐RS were obtained for the lactic acid:glycerol and propionic acid:glycerol DES, with molar ratios of 1.2 and 1.8, respectively (Table 1). On the other hand, the evaluation of the systems that presented higher activity coefficients, indicative of lower fucoidan solubility, revealed that the presence of proline played a relevant role in subsequent analyses. However, the glycerol:lactic acid DES emerged as the most promising when the COSMO‐RS results were combined with the experimental results. The trends predicted by COSMO‐RS were corroborated by experimental results in terms of extraction yield and chemical characterization of the extract.

The acidity of the medium is one of the main factors in determining the solubility of sulfated polysaccharides from macroalgae. The pH directly influences the ionization of the functional groups present in the biopolymers and can impact the solvent interaction of the polysaccharide from the cell matrix itself [36, 37]. Therefore, the analysis of the pH of eutectic solvents is important to understand their ability to extract sulfated compounds from *S. muticum*. In addition, this polysaccharide has a relatively low pKa; therefore, the choice of DES with a pH lower than these values will result in a more efficient strategy regarding selective extraction [38, 39]. Fucoidan has pKa values around 1.0 and 2.5 [40]. Thus, extraction efficiency is based on media where the pH is lower than the pKa of fucoidan, as it results in the protonation of the carboxyl and sulfate groups, which increases its solubility in polar environments [40]. Unlike the traditional method that uses hydrochloric acid, DESs with an acidic character and a moderately acidic pH (0.4 to 2.7), provide less aggressiveness to the structure of the extracted compound. DESs such as 12PDO:AcOH (pH 0.83), LacAc:Gly (pH 0.87), and PropAc:Gly (pH 0.54) proved satisfactory for the extraction of fucoidan (Table 1).

In addition, DESs rich in hydroxyls facilitate the formation of hydrogen, allowing greater interaction with polysaccharides, essentially those formed by polyols, such as glycerol, 1,2‐propanediol and trimethylolpropane, which favors the formation of hydrogen bond networks [10, 39]. As in Wang [41], who evaluated different types of polyol‐based eutectic solvents, for the breakdown of the hydrogen structure of biomass and the solubilization of the material of interest. These noncovalent interactions favor the solubilization of polysaccharides, as they resemble the behavior of water, in which they provide a hydrophilic environment, favoring the extraction and solubilization of the polysaccharide [10, 42]. Thus, the balance between acidity and solvation capacity is crucial for the extraction process (Table 1).

The thermal degradation of DESs is a critical parameter for use in extractive processes that involve heating. Thermogravimetric analysis (TGA) is capable of defining the thermal limit at which the solvent begins to degrade, which can compromise efficacy, generate by‐products and modify the structure of desirable compounds [43, 44]. Thus, differential thermal analysis (Td (°C)) for each DES is of utmost importance, as it ensures the integrity of the extraction process. Before process optimization, the extraction temperature was maintained at 60°C for 2 h, so DESs with Tda higher than this temperature were considered suitable for maintaining process stability. DESs such as 14BDO:GlycAc with a Td of 50°C, AcOH:Gly with a Td of 61°C, AcOH:TMP with a Td of 63°C and PropAc:Gly with a Td of 69°C showed decomposition temperatures very close to or lower than that of the initial extraction (Table 1). As a result, they can compromise the process, especially in extractions with longer times and constant agitation, enabling the formation of by‐products, such as volatile carboxylic acids and oxidative compounds, which interfere with the extraction selectivity. In addition, the loss of chemical stability can interfere with the reuse of these DESs, rendering their use in constant processes unfeasible [45].

Other eutectic solvents, such as LacAc:Gly with a Tda of 218°C, LacAc:TMP with a Tda of 229°C, 13BDO:GlycAc with a Tda of 188°C, and 12PDO:GlycAc with a Tda of 128°C, provide excellent thermal resistance and can withstand not only the conditions of the extraction process but also possible temperature elevations on a pilot or industrial scale (Table 1). The high Tda value is related to the presence of components that present low volatility, a higher density of hydroxyl groups, and the presence of branched chains, delaying thermal degradation [46, 47]. It can be observed that glycolic acid‐based DESs present high thermal degradation, which can be related to their more robust hydrogen bonding networks, increasing the stability of the eutectic solvent matrix [48, 49]. Therefore, the decomposition temperature is crucial, not only for the selection of compatible DES, but also as an indicator for reuse in multiple extraction cycles.

### 2.3 Extraction Fucoidan

#### 2.3.1 Yield and Chemical Composition of Fucoidan

**FIGURE 2:**
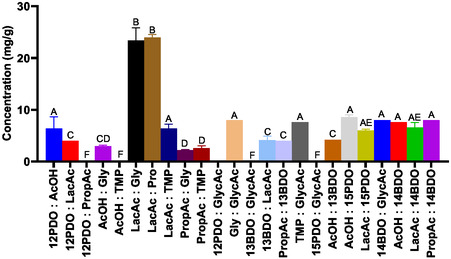
Comparison of fucoidan yields (mg FUC/g AS) of eutectic solvents: 1,2‐Propanediol: Acetic acid (12PDO:AcOH); 1,2‐Propanediol:Lactic Acid (12PDO:LacAc); 1,2‐Propanediol:Propionic acid (12PDO:ProAc); Acetic acid:Glycero (AcOH:Gly); Acetic acid:Trimethylolpropane (AcOH:TMP); Lactic Acid:Glycerol (LacAc:Gly); Lactic Acid:Proline (LacAc:Pro); Lactic Acid:Trimethylolpropane (LacAc:TMP); Propionic acid:Glycerol (PropAc:Gly); Propionic acid:Trimethylolpropane (PropAc:TMP); 1,2‐Propanediol:Glycolic acid (12PDO:GlycAc); Glicerol:Glycolic acid (Gly:GlycAc); 1,3‐Butanediol:Glycolic acid (13BDO:LacAc); 1,3‐Butanediol:Lactic acid (13 BDO:LacAc); Propionic acid:1,3‐Butanediol (PropAc:13 BDO); Trimethylolpropane:Glycolic acid (TMP : GlycAc); 1,5‐Pentanediol:Glycolic acid (15PDO:GlycAc); Acetic acid:1,3‐Butanediol (AcOH:13BDO); Aceticacid:1,5‐Pentanediol (AcOH:15PDO); Lactic acid:1,5‐Pentanediol (LacAc:15PDO); 1,4‐Butanediol:Glycolic acid (14BDO:GlycAc); Acetic acid:1,4‐Butanediol (AcOH:14BDO); Lactic acid:1,4‐Butanediol (LacAc:14BDO); and Propionic acid:1,4‐Butanediol (PropAc:14BDO). Statistical significance was set at *p* < 0.05. Distinct capital letters indicate significant differences between the experimental groups.

The effectiveness of eutectic solvents in the extraction of fucoidan from *S. muticum* cell walls is shown in Figure 2. This figure shows fucoidan extraction yields (mg of fucoidan/g of dry seaweed) through the initial process at 60°C for 2 h. Therefore, the properties of each eutectic solvent determined the efficiency of the extraction of the sulfated polysaccharide, influencing the solubilization of the seaweed matrix and the selective release of the compound.

The yield results of fucoidan from *S. muticum* varied widely, which may indicate that there was an influence of the chemical composition of each eutectic solvent on the cell wall of the algae. The highest yields were observed for the compounds LacAc:Gly and LacAc:Pro, with concentrations higher than 23 mg/g of dry algae, and presented statistical differences between the other solvents. As can be seen in Figure 1, fucoidan yielded 10.9% (w/w) using conventional acid extraction. Although fucoidan extracted with eutectic solvents did not show higher yields, the results obtained were satisfactory and within the expected range. Conventional extraction methods generally offer limited control over parameters such as acidity and solvent/biopolymer interactions, which can lead to the simultaneous extraction of other algal components. In contrast, DES systems form dense networks of hydrogen bonds and exhibit adjustable acidity and polarity, promoting effective cell wall disruption and increasing extraction selectivity [10, 39]. This characteristic is particularly advantageous for applications requiring higher purity fucoidan.

In addition, the efficiency of the two DESs (LacAc:Gly and LacAc:Pro) can be attributed to their ability to break down the cell wall of algae and solubilize larger amounts of compounds. The authors James et al. presented yields ranging from 12.9 to 22.24 g/100 g of algae [50]. In this study, the authors used techniques such as microwaves, thermochemistry, ultrasonics, and supercritical water, with the use of choline chloride with glycerol. DES based on choline chloride with glycerol is widely used for the extraction of sulfated polysaccharides, because of their good solvation capacity and thermal stability. However, it can be seen that the DES, LacAc:Gly, being free of halides, containing only organic acid and polyol, presented comparable efficiency, indicating the possibility of replacing ChCl, since, although it is a biodegradable DES, it presents toxicity at certain concentrations.

In contrast, the other eutectic solvents presented intermediate yields, between 7 and 11 mg/g, namely 12PDO:GlyAc, 13BDO:GlyAc, Gly:GlyAc and 14BDO:GlyAc. Although GlyAc has acidic and hydrophilic properties, the extraction yield was not high compared to LacAc. This factor may be related to lower thermal stability and lower intermolecular interaction with the compounds present in the cell wall [51, 52]. Alcohols such as 12PDO and 14PDO favor hydroxyl groups in the formation of hydrogen bonds; however, their carbonic elongation reduced polarity, making it difficult to rupture the cell matrix [10, 39]. The DESs AcOH:TMP, PropAc:Gly, and PropAc:TMP showed the lowest yields (<6 mg/g). The acidity of these acids, being weak, did not favor the protonation of the anionic groups of fucoidan and other compounds present in the cell wall of the macroalgae [53]. Furthermore, TMP, being a trifunctional molecule, that is, with a larger spherical volume, did not present good mobility and interaction with the algae [54].

The chemical composition of the fucoidan obtained by extractions with DESs may indicate the selectivity and efficiency of these solvents in the solubilization of fucoidan from the cell wall of *S. muticum*. Table 2 presents the chemical composition of the extracts obtained from each extraction based on the total sugar, uronic acid, protein, polyphenol and sulfate content.

**TABLE 2:** Chemical composition of fucoidan obtained by each eutectic solvent. Statistical data were considered significant at *p* < 0.05. Distinct capital letters indicate significant differences between experimental groups.

Eutectic solvents	Total sugar, %	Uronic acid, %	Protein, %	Polyphenois, %	Sulfate, %
12PDO:AcOH	17.79 ± 0.09^A^	4 ± 0.04^A^	0.84 ± 0.0^A^	0.10 ± 0.01^A^	7.63 ± 0.10^A^
12PDO:LacAc	12 ± 0.03^B^	2 ± 0.02^B^	2.3 ± 0.02^B^	0.08 ± 0.01^B^	3.49 ± 0.05^B^
12PDO:PropAc	12 ± 0.01^B^	3 ± 0.00^C^	0.67 ± 0.01^C^	0.21 ± 0.00^C^	0.00 ± 0.0^C^
AcOH:Gly	11 ± 0.08^C^	2 ± 0.01^B^	0.43 ± 0.01^D^	0.08 ± 0.01^B^	11.65 ± 0.15^D^
AcOH:TMP	5 ± 0.08^D^	1 ± 0.02^D^	0.04 ± 0.00^E^	0.04 ± 0.00^D^	0.00 ± 0.0^C^
LacAc:Gly	16 ± 0.10^E^	5 ± 0.04^E^	0.83 ± 0.01^A^	0.10 ± 0.01^A^	9.35 ± 0.03^E^
LacAc:Pro	12 ± 0.03^B^	8 ± 0.02^F^	0.83 ± 0.01^A^	0.10 ± 0.0^A^	4.12 ± 0.02^F^
LacAc:TMP	7 ± 0.01^F^	2 ± 0.01^B^	2.99 ± 0.01^F^	0.07 ± 0.01^EB^	0.55 ± 0.0^G^
PropAc:Gly	14 ± 0.01^G^	2 ± 0.01^B^	1.85 ± 0.01^G^	0.11 ± 0.01^A^	9.32 ± 0.0^H^
PropAc:TMP	9 ± 0.03^H^	2 ± 0.02^B^	2.45 ± 0.01^H^	0.08 ± 0.01^B^	0.46 ± 0.01^I^
12PDO:GlycAc	8 ± 0.03^I^	2 ± 0.03^B^	4.44 ± 0.02^I^	0.03 ± 0.01^FD^	0.00 ± 0.0^C^
Gly:GlycAc	26 ± 0.13^J^	8 ± 0.18^F^	7.12 ± 0.08^J^	0.24 ± 0.01^G^	2.19 ± 0.01^K^
13BDO:GlycAc	10 ± 0.01^K^	2 ± 0.01^B^	5.53 ± 0.00^K^	0.04 ± 0.00^D^	0.00 ± 0.0^C^
13BDO:LacAc	6 ± 0.03^L^	1.2 ± 0.01^G^	5.87 ± 0.02^L^	0.01 ± 0.01^H^	1.11 ± 0.02^L^
PropAc:13BDO	7 ± 0.05^F^	1.2 ± 0.04^G^	2.43 ± 0.03^M^	0.01 ± 0.01^H^	0.0 ± 0.0^C^
TMP:GlycAc	6 ± 0.04^L^	2 ± 0.02^B^	3.13 ± 0.02^N^	0.02 ± 0.01^FH^	12.13 ± 0.02^M^
15PDO:GlycAc	5 ± 0.02^D^	2 ± 0.03^B^	5.21 ± 0.04^O^	0.06 ± 0.00^IE^	0.0 ± 0.0^C^
AcOH:13BDO	6 ± 0.08^L^	1.1 ± 0.03^H^	5.08 ± 0.04^P^	0.01 ± 0.01^H^	1.41 ± 0.02^N^
AcOH:15PDO	7 ± 0.02^F^	3.3 ± 0.01^I^	16.58 ± 0.01^Q^	0.05 ± 0.01^JDI^	4.29 ± 0.06^O^
LacAc:15PDO	3 ± 0.01^M^	1.2 ± 0.01^G^	16.29 ± 0.01^R^	0.02 ± 0.01^FH^	3.24 ± 0.02^P^
14BDO:GlycAc	6 ± 0.01^L^	7.5 ± 0.06^J^	17.01 ± 0.01^S^	0.07 ± 0.01^BI^	5.10 ± 0.07^Q^
AcOH:14BDO	3 ± 0.04^M^	1 ± 0.01^D^	8.62 ± 0.12^T^	0.02 ± 0.01^DH^	0.16 ± 0.0^R^
LacAc:14BDO	10 ± 0.02^K^	3.3 ± 0.01^I^	17.18 ± 0.01^U^	0.05 ± 0.01^DIJ^	6.58 ± 0.07^S^
PropAc:14BDO	7 ± 0.04^F^	2.9 ± 0.01^K^	12.77 ± 0.06^V^	0.04 ± 0.01^DJ^	2.7 ± 0.03^U^

The composition of the total sugars varied among the DESs, with Gly:GlyAc having the highest content (26% of total sugars), showing a statistical difference from the other solvents. This can be explained by the combination of a hydroxylated polyol, such as glycerol, and a weak acid, such as glycolic acid, which promotes a strong degradation capacity of the algal cell wall matrix, resulting in the solubilization of the carbohydrates present [55, 56]. In addition, the DESs 12PDO:AcOH (17.79%) and LacAc:Gly (16%) also had a high carbohydrate content, and a statistical difference was observed between the two groups with the other DESs, where it can be observed that the combination of a diol with acetic acid or lactic acid, both with moderate acidity, was beneficial for the extraction of neutral and partially charged polysaccharides. On the other hand, the DESs TMP:GlycAc presented a sulfate content of 12.13%, AcOH:Gly with a content of 11.65% and LacAc:Gly with a content of 9.35%. These results suggest that these DESs, with hydrogen bond stabilization capacity and low viscosity, may be able to solubilize the fractions containing sulfates. TMP:GlycAc, because it presents a highly branched triol, was able to interact with the sulfated groups through simultaneous bonds, while glycolic acid was able to make the environment acidic, preventing the desulfation of fucoidan [54]. LacAc:Gly, because it has a high sulfate content, may be related to lactic acid, which provides moderate acidity and is capable of breaking the intermolecular bonds of the cell wall, and glycerol, which may favor the solubilization of the broken macromolecules of the cell wall. Some studies, such as Husni et al. and Shanthi et al. [57, 58], adopted traditional methods for the extraction and purification of the fucoidan molecules, using HCl and ethanol, and obtained sulfate contents ranging from 9.25% to 30.62%, showing that green extractions are as effective as traditional methods, denoted by high sulfate contents.

The protein content in the extracts may indicate a greater selectivity of these respective DESs, such as LacAc:14BDO with a content of 17.18%, 14BDO:GlycAc with a content of 17.01%, AcOH:15PDO with a content of (16.58%), and LacAc:15PDO with a content of 16.29%, even with high contents compared to the other DESs, these groups presented statistical difference between them. This effect may be related to the lower specificity of these solvents, which were able to solubilize only high levels of structural proteins in the cell wall. However, the presence of these high protein concentrations may compromise the purity of fucoidan, especially in in vitro assays [59, 60], which require additional purification steps. On the other hand, DESs such as AcOH:TMP, AcOH:Gly, 12PDO:PropAc, and LacAc:Gly presented low protein concentrations, being able to present greater selectivity for carbohydrates, as can be seen in Table 2, showing a significant difference between the DESs.

Polyphenols were present at low levels in all extractions, with higher concentrations in Gly:GlycAc (0.24%) and 12PDO:PropAc (0.21%). This may indicate that these DESs promoted a more efficient extraction of polysaccharides, with a low coextraction of phenolics. This selectivity may be beneficial for applications that wish to study and evaluate the biological effects attributed only to the sulfated polysaccharide (fucoidan). Furthermore, the presence of polyphenols helps in antioxidant and anti‐inflammatory properties [60]. In Obluchinskaya et al. and Liyanage et al. [60, 61], the authors studied the anti‐inflammatory effects of fucoidans, in which they were favored by the presence of polyphenols, which varied from 3.58% to 10.1%. The presence of these compounds, even in low concentrations, can be advantageous, especially in in vitro assays aimed at pharmaceutical applications, helping in cellular inhibition, antioxidant activity, regulation of inflammatory enzymes, and protection against oxidative stress [60, 61, 62].

For uronic acids, the highest levels were obtained in the DESs Gly:GlycAc and LacAc:Pro, both with levels of 8%, with no statistical difference between them. Since fucoidan is predominantly composed of sulfated fucose units and naturally contains low amounts of uronic acids [38], elevated uronic acid levels may indicate alginate coextraction. This is because the structural composition of alginate is mainly formed by mannuronic and glucuronic acid units [63]. Therefore, the higher levels of uronic acid observed for these DESs suggest lower selectivity toward fucoidan, potentially compromising the purity of the product and, consequently, its bioactive properties, such as biological activity, anti‐inflammatory, and anticoagulant effects, thus requiring additional purification steps [38, 64]. Eutectic solvents such as AcOH:Gly, TMP:GlyAc, AcOH:14BDO, and LacAc:Gly presented low levels of uronic acids in their structures, indicating higher purities in the solubilization of sulfated polysaccharides, in addition to not presenting a statistical difference between the DESs AcOH:Gly and TMP:GlyAc.

### 2.4 Optimization of Fucoidan Extraction

With the aim of identifying the most favorable conditions for fucoidan extraction from *S. muticum* using LacAc:Gly, the results of the central composite design provide relevant information on the influence and interaction of the evaluated process variables. Furthermore, the LacAc:Gly system was considered the most promising based on criteria such as yield, high sulfate content, low uronic acid content, and DES production cost (Table SA3). It stood out for its selectivity, demonstrating low uronic acid levels, indicating less alginate coextraction. Furthermore, the high sulfate content confirmed its efficiency in the extraction of fucoidan. Besides that, the low cost of DES formulation, estimated at $ 0.77 per 10 mL of solvent, reinforces its applicability on both laboratory and industrial scales (Table A4).

Furthermore, the experimental design consisted of 8 factorial points, 6 axial points, and 3 replicates at the central point, totaling 17 experiments. The coded levels of the variables were set at −1 and +1, with the axial points being (±*α*, 0, 0), (0, ±*α*, 0), and (0, 0, ±*α*). The *α* value was 1.682, allowing rotation of the experimental design with three factors, as shown in Table SA2. The study evaluated the influence of the variables water percentage (0%–20%), temperature (46°C–114°C), and *S*/*L* ratio (0.0719–0.1281 *S*/*L*) on the extraction of fucoidan from *S. muticum* using LacAc:Gly (1,20) (Table SA3).

Based on the experimental design studied and the responses obtained, desirability was used for multivariate optimization, integrating the different process objectives (Figure SA1). Although it presented limitations in adjusting some models, desirability proved effective in identifying the optimal operational condition. Therefore, this behavior is expected in multivariate processes, demonstrating that desirability helps in identifying the ideal system. In the present study, the models for yield (*R* ^2^ of 95.44% and adjusted *R* ^2^ of 0.8957) and hexuronic acid (*R* ^2^ of 0.8337 and adjusted *R* ^2^ of 0.6201) showed satisfactory predictive performance, while the models for hexose (*R* ^2^ of 0.4786 and adjusted *R* ^2^ of 0) and sulfate (*R* ^2^ of 0.5378 and adjusted *R* ^2^ of 0) showed low coefficients of determination. Nevertheless, the desirability model was able to propose an operating range aligned with the process objectives, demonstrating its role in supporting experimental decision‐making.

**FIGURE 3:**
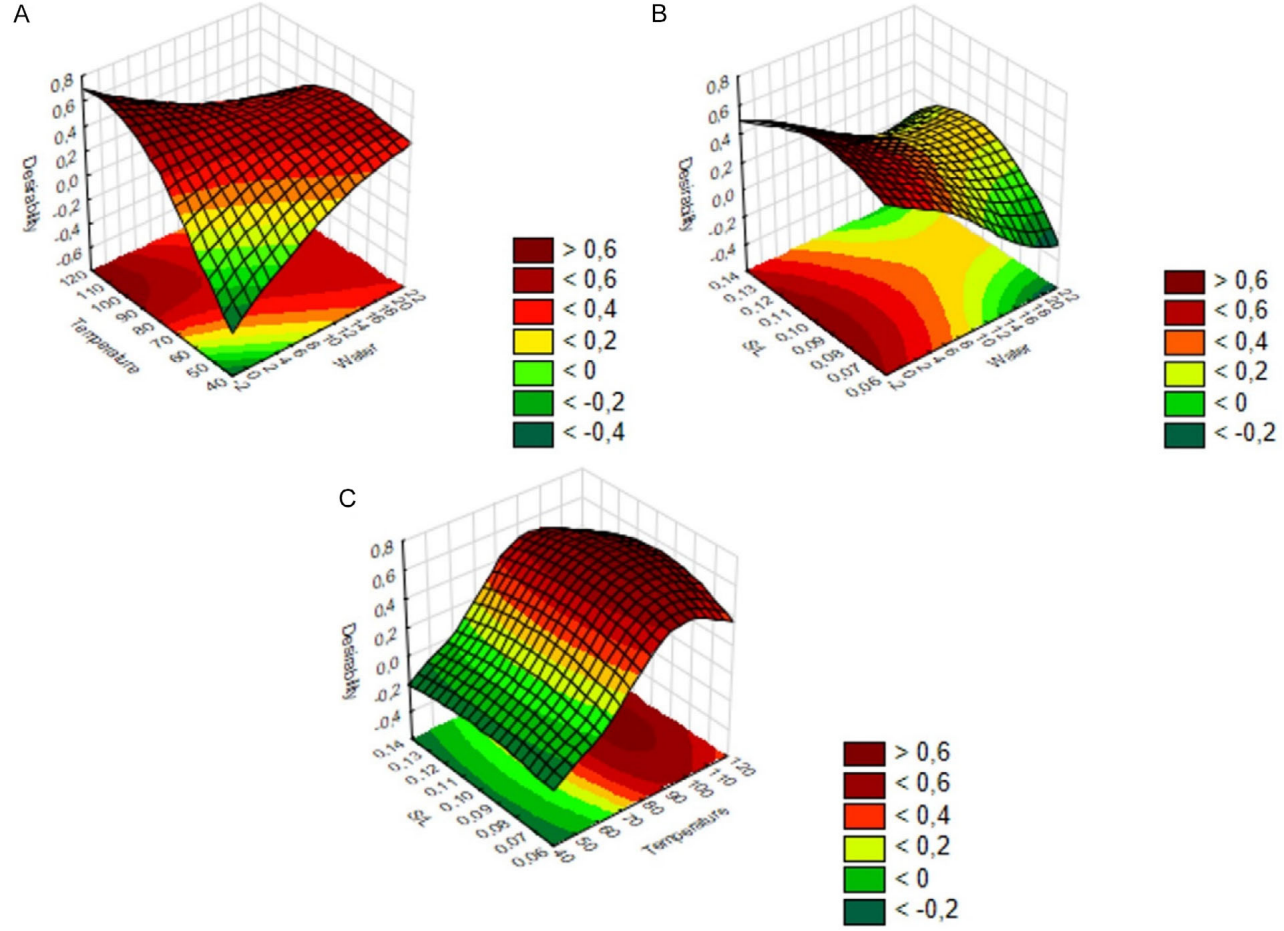
Desirability boundary surface generated by 560‐spline fitting method for the eutectic solvent AcLac:Gly (1,20). (A) Responses with temperature × water parameters; (B) Responses with S/L × water parameters; and (C) Responses with *S*/*L* × temperature parameters.

Pareto diagram analysis (Figure SA2) showed that temperature was the most determinant factor in the model developed for fucoidan extraction, significantly influencing the yield and hexuronic acid content, with linear and quadratic effects observed. The interaction between temperature and *S*/*L* ratio (2Lby3L) was significant for hexuronic acid. Furthermore, linear and quadratic interactions were not removed from the model, as their *R* ^2^ and adjusted *R* ^2^ decreased, compromising the model studied. Using boundary surface analysis and desirability, the optimal conditions for fucoidan extraction with the DES LacAc:Gly (1.20) were identified. The optimum point provided by the optimization using Statistica software was 0% water, a temperature of 106°C, and an *S*/*L* ratio of 0.089, in order to maximize the yield and purity of the extracted material (Figures 3 and SA1).

From the model, the optimal parameters provided yield the best results, with a yield of 0.1494 mg/mg, hexose (total sugars) content of 0.2614 mg/mg, hexuronic acid content of 0.017 mg/mg, and sulfate content of 0.170 mg/mg (Figure SA1). However, the experimental results were lower than expected, with a yield of 0.089 mg/mg, hexose content of 0.160 mg/mg, hexuronic acid content of 0.025 mg/mg, and sulfate content of 0.155 mg/mg. Although the desirability model indicated parameters that would result in higher yield, hexose, and sulfate values, and lower uronic acid contents, the opposite was observed in the experimental study. However, it provided an ideal range for maximizing these values.

Furthermore, the influence of high temperature (106°C) on the experimental results may explain the lower performance than predicted by the experimental model, even indicating maximization of yield, as obtained. Temperatures above 100°C can cause thermal degradation of functional groups that are more sensitive to extreme temperatures, favoring depolymerization reactions and can convert monosaccharides into byproducts, resulting in low levels of total available sugars and sulfates [65, 66, 67]. Furthermore, the absence of water in the DES can reduce extraction efficiency, since, in moderate proportions (below 30%) [68], it can stabilize the structure of fucoidan, reducing thermal degradation and viscosity of the medium [68, 69, 70]. Therefore, the combination of high temperature and total absence of water may have compromised the structure of the bioactive compound, which can be explained by the lower results of the experiments compared to those estimated by the predicted model. Although high temperatures maximize yields, this may indicate degradation of the elements and may also solubilize higher levels of components associated with fucoidan impurities, such as alginate and proteins [65, 71]. However, experimental order condition 2 was selected among the 17 trials of the experimental design, with parameters of 4% water, a temperature of 60°C, and 0.117 *S*/*L* (Table SA3). These parameters resulted in a yield of 0.066, 0.1758 mg/mg of hexose, 0.023 mg/mg of hexuronic acid, and 0.170 mg/mg of sulfate (Table SA5). Although the yield was below the value predicted by the statistical model, the choice of these parameters was related to the purity of the fucoidan. Furthermore, the extract contains a sulfated polysaccharide, with its biological activities related to the sulfate content present [64, 72].

The *S*/*L* ratio of 0.117 may indicate a balance in the extraction process. Very low *S*/*L* ratios lead to highly dilute systems, reducing mass transfer efficiency and solid‐liquid contact, which negatively affects extraction performance. On the other hand, very high proportions can lead to saturation of the medium, making it difficult to solubilize the compounds, making the extraction viscous [73]. In addition, the low content of hexuronic acids, it may indicate lower alginate contaminants in the sample, being favorable for applications with greater selectivity [65, 71]. The moderate temperature and presence of water are within the desirability surface (Figure 3), favoring the preservation of the groups present in the fucoidan structure, without thermal degradation.

### 2.5 Physicochemical and Structural Characterization of Fucoidan

**FIGURE 4:**
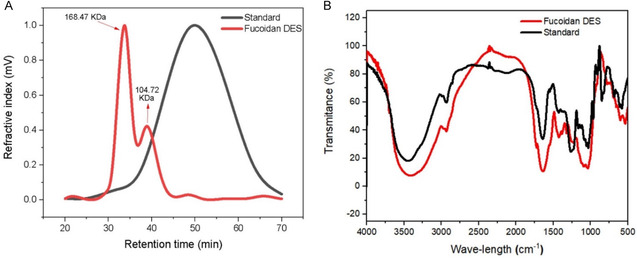
(A) Molecular weight distribution of fucoidan from *S. muticum.* (B) FT‐IR analysis of polysaccharides extracted from *S. muticum* in the range of 500–4000 cm^−1^. Fucoidan DES (polysaccharide extracted by LacAc:Gly) and standard (polysaccharide provided by Sigma‐Aldrich).

Analysis of the molecular weight of the sulfated polysaccharide is of utmost importance to evaluate the structure and bioactive potential of the material, since the binding capacity of cellular receptors and pharmacological activities may be related to the molecular weight of the polysaccharide. Therefore, to evaluate the molecular weight, size exclusion chromatography with a refractive index (RI) detector was used with heparin standards of different molecular weights (Figure 4A).

The results obtained in Figure 4A show fucoidan with a bimodal profile, that is, with two main peaks. The first peak was more intense, with a molecular weight of 168.47 kDa, while the second peak had a smaller peak, approximately 104.72 kDa (Table SA6). This heterogeneous profile is characteristic of macroalgal fucoidans, in which their structure can vary depending on the alga, seasons, extraction method, and physicochemical conditions [74, 75, 76]. Since the highest molecular weight peak had greater intensity, it can be understood that LacAc:Gly was efficient for the extraction of high molecular weight biopolymers, resulting in the preservation of glycosidic bonds, which may be related to the low hydrolytic strength of this DES, that is, it did not solubilize as many sulfated contents [33, 56]. However, the presence of low molecular weight fucoidan is of utmost importance. Studies such as Suprunchuk and Chen et al. evaluated that low molecular weight fucoidans (5 to 100 kDa) present promising biological effects, such as cell viability, proliferation and greater cell migration to the wound injury area [77, 78]. Although the 168.47 kDa content favors applications in biomaterials or extracellular networks, it is likely that the 104.72 kDa content is responsible for the responses of the cellular assays that will be observed later, such as the promising responses of invasion and colony formation. However, this heterogeneity in molecular weights may amplify effects on the healing action of fucoidan extracted by LacAc:Gly.

Not only the molecular weight, but also physicochemical analysis by Fourier transform infrared spectroscopy (FT‐IR) was performed to characterize the structure of fucoidan extracted from *S. muticum* using the DES LacAc:Gly (1.20). Figure 4B shows the spectral profiles of fucoidan DES (extracted material) and fucoidan standard (Sigma‐Aldrich). FT‐IR analysis allowed the analysis of the material extracted by LacAc:Gly (1.20) with commercially available fucoidan, and it was possible to analyze whether the extracted material presented structural groups typical of polysaccharides and whether the extraction was efficient (Table A7). Thus, as shown in Figure 4, both samples presented bands originating from sulfated polysaccharides; however, there were small differences between them. Standard fucoidan and the fucoidan extracted with DEs presented bands around 3400 cm^−1^, indicating stretching vibrations of hydroxyl groups (‐OH) attributed to fucoses and other carbohydrates. However, it can be observed that Fucoidan DES presented a wider band in this peak, when compared to standard fucoidan, which may indicate moisture retention or even the presence of impurities such as DES hydroxylates. However, additional purification steps, such as membrane purification or ethanol washing procedures, could further reduce these impurities. The authors Banjare et al. and Koigerova et al. demonstrated that extractions with DES that compose glycerol can present, in this peak, more accentuated bands due to small interferences of the O=H stretching [55, 79]. The band present at 2900 cm^−1^ is related to the vibrations of methyl groups (‐CH) of aliphatic groups of the pyranoid rings, which were observed in both samples.

The band at 1640 cm^−1^ can be observed with greater intensity in the DES sample, which is attributed to the C=O vibrations of carboxylic or acetyl groups, such as hexuronic acid, guluronic acid and mannuronic acid residues. Although both spectra identified this peak, DES contained greater evidence, which may represent a higher percentage of impurities in fucoidan, such as alginate. The band at 1420 cm^−1^ is attributed to carboxyl vibrations (‐COOH), which are more intense in fucoidan DES, corresponding to hexuronic acid residues. However, the band at 1220–1250 cm^−1^ is related to asymmetric vibrations of sulfate groups (S=O), a characteristic group of fucoidan, with a greater intensity noted in the commercial sample. Although both presented this peak, the DES sample presented a lower degree of sulfation, which may be related to partial degradation in the extraction process. The band at 1030 cm^−1^ was associated with glycosidic symmetric vibrations (C=O=C), being associated with bond elongation in the pyranose ring, present in both samples. The band present at 840 cm^−1^, can be observed in the vibrations of the sulfated fucopyranose rings (C=O=S), being observed in both samples. Although Fucoidan DES did not present spectral structures related to the sulfate polysaccharide, with intensity comparable to the commercial one, spectra were identified that proved the structure of fucoidan.

**FIGURE 5:**
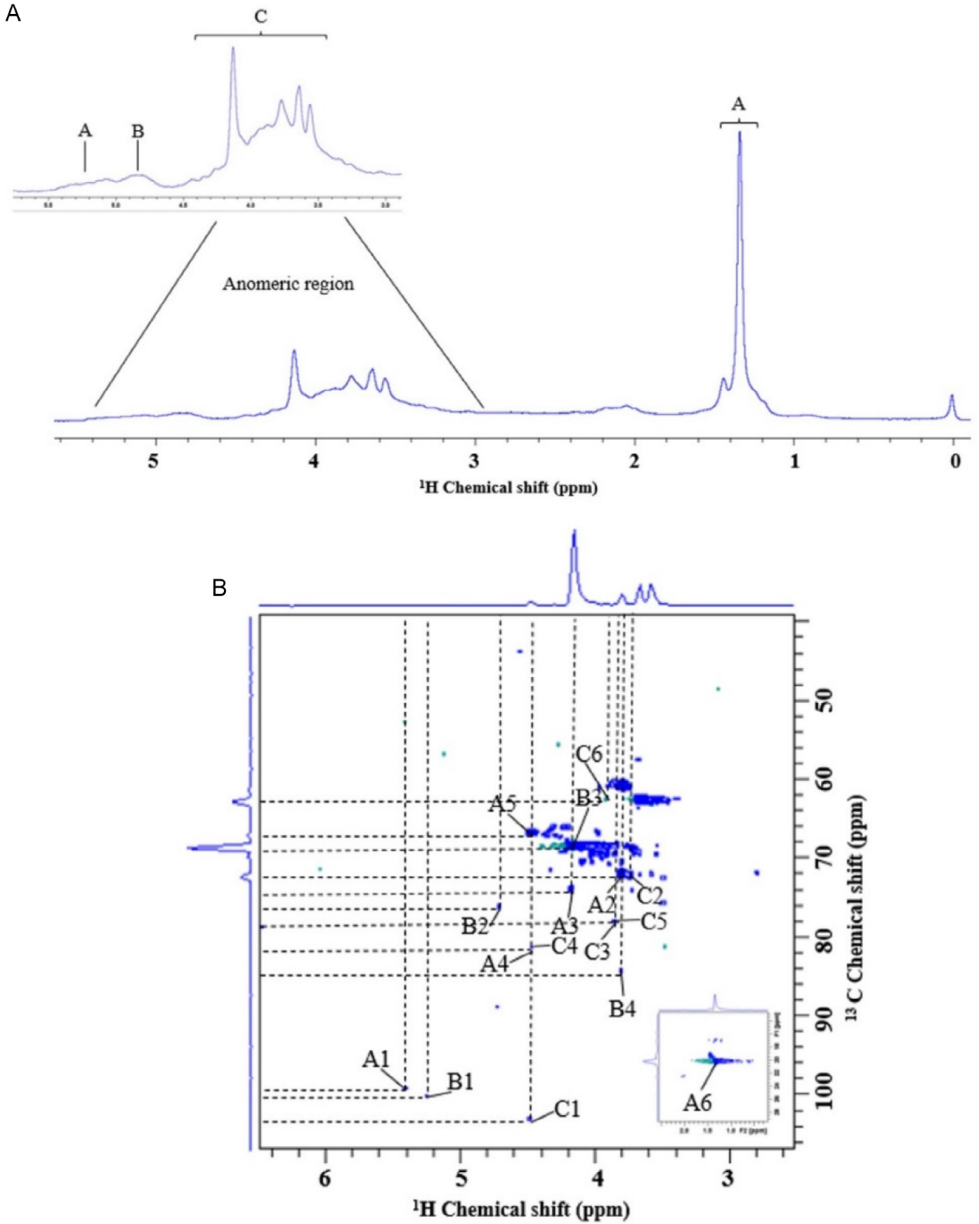
(A) ^1^H NMR spectrum highlighting the anomeric regions of fucoidan (DES extract). (B) HSQC spectrum (^1^H=^13^C) of fucoidan extracted with DES.

However, since FT‐IR primarily provides qualitative information about functional groups, a more detailed structural elucidation of the fucoidan extracted with the eutectic solvent required complementary analyses. Therefore, one‐dimensional ^1^H and two‐dimensional HSQC (^1^H=^13^C) spectra (Figure 5). This analysis allowed the identification of the constituent monosaccharides and the characterization of glycosidic bonds, branches, and the possible presence of methyl and acetyl groups in the sample.

**FIGURE 6:**
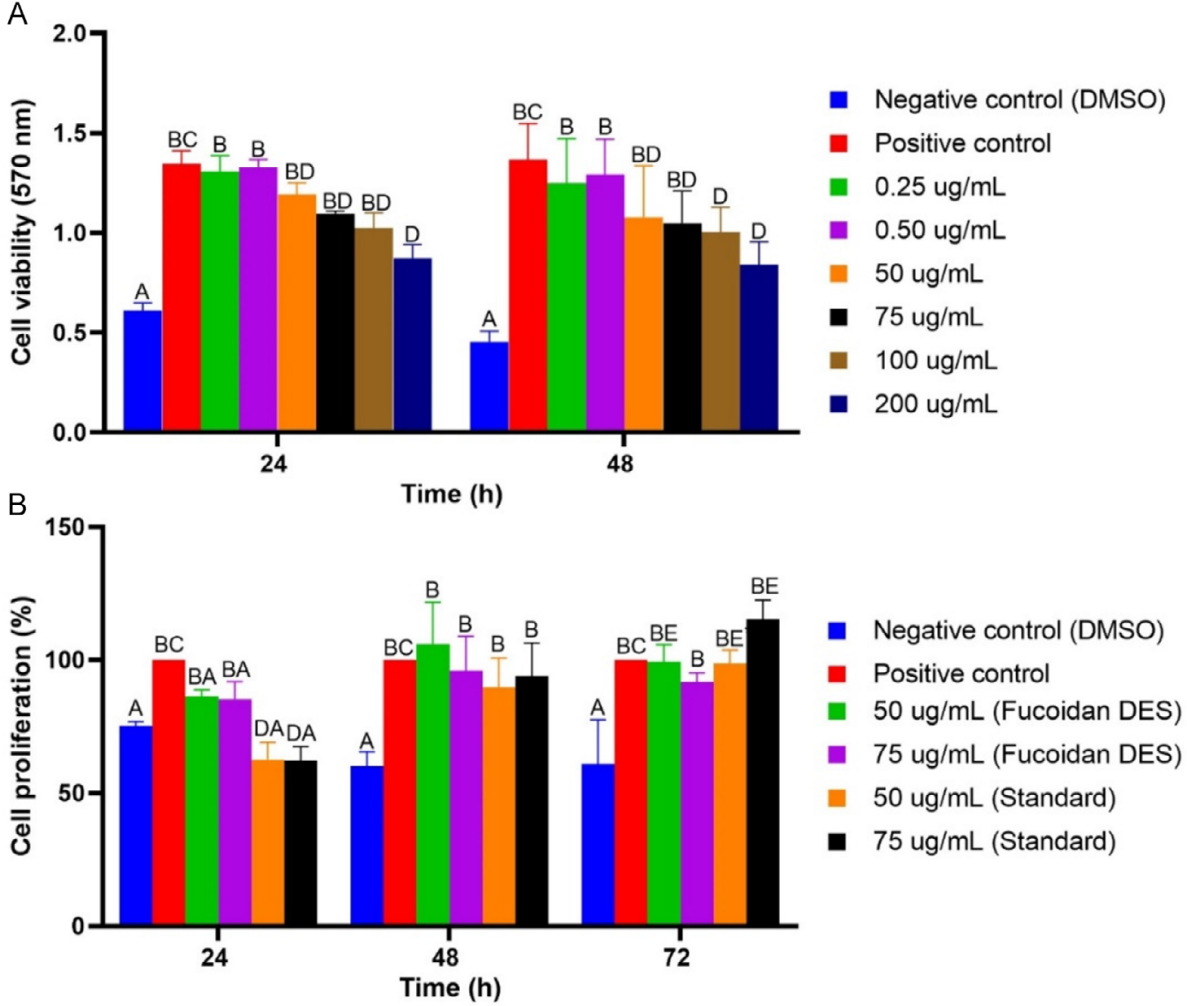
(A) Cell viability of fibroblasts after 24 and 48 h of exposure to fucoidan extracted with DES (LacAc:Gly) at different concentrations (0.25–200 μg/mL). (B) Cell proliferation of fibroblasts after 24, 48, and 72 h of exposure to fucoidan extracted with DES (LacAc:Gly) and Sigma fucoidan standard at concentrations of 50 and 75 µg/mL. Statistical data were considered significant at *p* < 0.05. Distinct capital letters indicate significant differences between experimental groups.

Structural characterization of the biomolecule extracted by LacAc:Gly (1.20) and an extension literature review allowed the analysis of the monosaccharide constituents and the pattern of glycosidic bonds present. Thus, the anomeric region of the 1H proton spectrum (Figure 5A) indicated three main signals, in residues A, B e C, approximately δ 4.5–5.5 ppm, indicating different types of sugar constituents [80, 81]. These signals were attributed to residues → 3,4)‐*α*‐L‐Fucp‐(1→ (residue A), L‐Fucp (residue B) and →6)‐*β*‐D‐Galp‐(1→ (residue C) (Table A8). The chemical shifts that can be observed (Figure 6B) for each of these residues are, δH 5.41 and δC 98.7 ppm (residue A), δH 5.23 and δC 99.9 ppm (residue B) and δH 4.48 and δC 102.9 ppm (residue C), normally found in fucoidan structures from brown algae [80]. Furthermore, it can be seen that the fucoidan presented a heterogeneous structure, as can be observed both in FT‐IR and in molecular weight retention times, presenting several types of glycosidic bonds, resulting in a varied and complex structure, commonly found in fucoidans.

The L‐fucose residues, mainly in the formation of *α*‐L‐Fucp, indicate that the extraction through DES was efficient in the solubilization of these monosaccharides. This residue observed in δH 1.37 ppm and δC 19.65 ppm (Table SA8), is characteristic of methyl groups with L‐fucose terminals, indicating that it presents a high concentration of this monosaccharide, since there is intense signaling, in addition to the other shifts that were from H2 to H5, δH 1.37–5.41 ppm and δC 66.6–98.7 ppm [80, 81, 82, 83]. This constituent is of utmost importance for fucoidan, as these methylated groups aid in the bioactivity of the molecule, especially in its anti‐inflammatory, antioxidant, and healing properties [64, 84]. Furthermore, since this peak presents strong evidence, it may indicate that there was no degradation or chemical modification after extraction with the solvent. Together with the L‐Fucp residues, with their δH 3.81–5.23 ppm and δC 68.3–99.0 ppm shifts, which may indicate additional branches

The *β*‐D‐galactose residue, with its *β*‐1→6 bonds, is essential for fucoidan, as galactose residues provide greater antiviral and immunomodulatory activity [83]. The chemical shifts present in this residue are δH 3.85–4.48 ppm and δC 62.3–102.9 ppm, being compatible with the chemical structure of galactose *β*‐pyranose. It can be understood that the displacement of carbon C6 at δ 62.3 ppm is typical of galactose with branching at position 6, which has a characteristic that grants greater flexibility and formation of three‐dimensional networks [80, 85, 86]. The combination of the structure of fucose and galactose presents a branched conformation, being compatible with fucoidans from macroalgae, such as *S. muticum*, and may indicate a sulfated polysaccharide with biological potential [64, 84]. Therefore, the analysis of the chemical shifts observed in Figure 5A and B shows that it presents a highly branched polysaccharide, with the residues L‐fucose, *β*‐D‐galactose and some acetylations, as main residues.

### 2.6 Antioxidant Activity

Analysis of the antioxidant activity of fucoidan extracted by DES LacAc:Gly (1.20) from *S. muticum* was important to evaluate of its potential as a neutralizing agent for reactive oxygen species (ROS). Therefore, three antioxidant assays were performed: ferric reducing antioxidant power (FRAP), 2,2‐diphenyl‐1‐pricylhydrazyl (DPPH), and 2,2′‐azino‐bis(3‐ethylbenzothiazoline‐6‐sulfonate) (ABTS). The quantification of antioxidant capacity was expressed as inhibition percentages, as shown in Table 3, using gallic acid as a standard.

**TABLE 3:** Measurement of antioxidant activities of fucoidan extracted *S. muticum* and gallic acid standard ferric reducing antioxidant power (FRAP), 2,2‐diphenyl‐1‐pricylhydrazyl (DPPH), and 2,2′‐azino‐bis(3‐ethylbenzothiazoline‐6‐sulfonate) (ABTS) of fucoidan obtained using optimized DES LacAc:Gly (1.20). Significant difference in all experimental groups with *p* < 0.05. Distinct capital letters indicate significant differences between experimental groups.

Sample	Concentration, μg/mL	ABTS, %	DPPH, %	FRAP, μg/μg dry weight
Fucoidan DES	0.08	6.42 ± 0.02^A^	14.06 ± 0.04^A^	230 ± 0.02^A^
	0.17	7.75 ± 0.02^B^	17.84 ± 0.03^B^	300 ± 0.04^B^
	0.33	17.15± 0.01^C^	21.54 ± 0.01^C^	420 ± 0.14^C^
	0.67	25.84 ± 0.01^D^	30.45 ± 0.02^D^	690 ± 0.33^D^
Gallic acid	0.5	29.47 ± 0.01^E^	15.32 ± 0.03^E^	150 ± 0.03^E^
	1	36.32 ± 0.04^F^	26.74 ± 0.02^F^	310 ± 0.02^F^
	1.5	42.35 ± 0.05^G^	34.92 ± 0.06^G^	440 ± 0.17^G^
	2.0	51.02 ± 0.02^H^	46.59 ± 0.04^H^	610 ± 0.06^H^

Analysis of the results revealed a concentration‐dependent relationship, where increasing the concentration of fucoidan resulted in a greater inhibitory effect. Among the methods performed, FRAP presented the highest inhibition levels, with approximately 690 μg**/**μg of inhibition at a concentration of 0.67 μg/mL, followed by DPPH with approximately 31% and ABTS with 26% inhibition. Yue et al. [87], evaluated the antioxidant activity of fucoidan extracted from brown algae using FRAP and ABTS assays, obtaining inhibition values ranging from 1.15 to 4.50 mg Trolox/g of fucoidan. The authors observed greater antioxidant activity in the FRAP assay compared to ABTS, suggesting that this difference may be associated with structural characteristics of the polysaccharide, such as the monosaccharide composition. Besides that, Kim et al. [88] evaluated the antioxidant effects of extracts obtained from *Sargassum* sp., obtaining results for ABTS, DPPH, and FRAP of 1.0, 6.50 mg/mL and 18.8 mg of gallic acid/g of extract, respectively, obtaining a higher content in the FRAP and DPPH tests, as seen in this study. In addition, fucoidan extracted by DES, at a concentration of 0.67 μg/mL, showed relevant results of antioxidant activity. Although the gallic acid standard, at a concentration of 2.0 μg/mL, exhibited inhibitions of 51%, 47%, and 610 μg/μg in the ABTS, DPPH, and FRAP assays, respectively, the performance of fucoidan was considered significant. These results indicate that the functional structures identified in the NMR and FT‐IR spectra (Figures 4B and 5A), associated with the presence of sulfated groups in the bioactive, contributed decisively to its antioxidant activity.

In this context, the distribution of sulfate groups along the fucoidan chain can act as active sites capable of stabilizing metal ions, favoring the reduction of Fe^3+^ [64]. Additionally, these sulfate groups can interfere with intramolecular and intermolecular hydrogen bonds, facilitating hydrogen donation and, consequently, enhancing the reducing activity mechanisms of the polysaccharide [89]. Compared with the values reported in the literature [87, 88], the inhibition levels obtained were within the expected range, as the macroalgae underwent pretreatment, removing percentages of polyphenols and pigments present. In addition, the increase in the FRAP assay results may indicate that the selected DES favored the solubilization of fucoidan with a large number of reducing groups. However, the lack of phenolic groups and pigments such as phlorotannins and carotenoids may have resulted in low responses to DPPH^•^ and ABTS^•+^ free radicals [90, 91, 92]. Thus, it was observed that the extracted fucoidan has significant antioxidant capacity, which is essential in environments where there is oxidative stress, such as wounds and inflammation.

### 2.7 In Vitro Cellular Assays

In vitro cytotoxicity assays are an important tool for early identification of the potential cytotoxicity of bioactive compounds during the initial screening phases. Although they do not fully replicate the cellular complexity of an organ or tissue, these assays provide essential preliminary data to support the selection of candidate molecules for progression to more advanced stages of preclinical evaluation. In the case of brown macroalgae, such as *S. muticum*, this analysis is of paramount importance, as this species can accumulate elements such as arsenic and other potentially toxic elements [29]. Thus, the MTT assay allows for an analysis of the biocompatibility of fucoidan.

Therefore, the viability of L929 fibroblast cells was evaluated in the presence of standard concentrations (0.25, 0.5, 50, 75, 100, and 200  µg/mL) of fucoidan DES extracted from *S. muticum* using the MTT assay (Figure 6A). In this assay, as in all subsequent assays, 5% DMSO was used as a negative control. The results obtained (Figure 6A) indicated that fucoidan DES was nontoxic to L929 fibroblast cells at concentrations up to 75 µg/mL, when compared to the positive control. Although lower concentrations (0.25 and 0.5 µg/mL) were also nontoxic, they may not provide adequate biological activity.

Thus, concentrations of 50 and 75 µg/mL were selected for subsequent experiments. Consistent with the results of this study, Wu et al. evaluated the cytotoxic effect of low molecular weight fucoidan on primary fibroblast cell lines, incubating them for 24 and 48 h at concentrations ranging from 10 to 75  µg/mL [12]. The authors observed that at these concentrations, the molecule was viable for the cells, with no significant difference between the groups, in which minimum concentrations were used for biological activity, as in this study.

At concentrations of 100 and 200 µg/mL, it was possible to observe that there was a significant decrease in cell viability, indicating that at concentrations greater than 100 µg/mL, fucoidan becomes toxic to cells. However, the toxicity at high concentrations of fucoidan extracted by LacAc:Gly may be associated with the presence of impurities that may have been extracted in the processes, such as solvent residues and uronic acids, which at high concentrations may compromise cell viability [71, 93]. The authors Dörschmann et al. evaluated the cell viability of fucoidan from different macroalgae in melanoma cell lines, in which they observed that such fucoidans did not present a toxic effect on the cells, being viable at concentrations of 100 μg/mL, which may be related to a fucoidan with a higher percentage of purity [93]. Although fucoidan is a polysaccharide with low toxicity to cells, studies indicate that its bioactivity can be influenced by the degree of sulfation and the presence of contaminants, acting negatively on cells [89, 94]. This can be explained by the structure of fucoidan (Figure 4B), where the bioactive substance indicates the presence of contaminants such as hexuronic acid and a low degree of sulfation.

Furthermore, the cell proliferation assay is commonly used to assess whether a bioactive compound can inhibit or stimulate the growth of healthy cells, such as fibroblasts [95]. Therefore, the effect of fucoidan extracted using LacAc:Gly on the proliferation of L929 fibroblast cells was evaluated using the MTT assay at different incubation times (24, 48, and 72 h) in the presence of 50 and 75 µg/mL Fucoidan DES. Sigma's fucoidan was used for comparison in this and all subsequent experiments (Figure 6B).

At all times, Fucoidan DES and Sigma's Fucoidan demonstrated lower proliferative effects. At 24 h, for example, Fucoidan DES inhibited proliferation by approximately 13% and 14% at concentrations of 50 and 75 µg/mL, respectively. Sigma's Fucoidan inhibited approximately 37% of the cells, respectively [67, 96, 97, 98]. At the other time points, Fucoidan DES showed a better proliferative effect when compared to Sigma's Fucoidan, especially at 48 h, approximately 5% at a concentration of 50 µg/mL [93, 99]. Although Fucoidan DES did not demonstrate a significant effect on increasing cell proliferation, these results corroborate its nontoxic activity profile.

**FIGURE 7:**
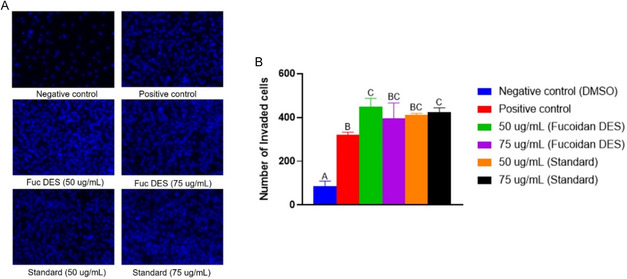
Evaluation of cell invasion after treatment with fucoidan at different concentrations (50 and 75 µg/mL). (A) Images of L929 cell invasion using Matrigel with different treatments. (B) Quantification of the number of invading cells under different treatment conditions. Statistical data were considered significant at *p* < 0.05. Distinct capital letters indicate significant differences between experimental groups.

Furthermore, fibroblasts are among the most important cells in the wound healing microenvironment. In this context, they contribute to the remodeling of the extracellular matrix through the secretion of proteins such as collagen and fibronectin, as well as cytokines, which aid in the wound healing process [100]. Therefore, the study aimed to evaluate the role of fucoidan DES in the invasion of L929 fibroblasts using a Transwell assay (Figure 7).

The results obtained using fucoidan DES showed superior invasion performance, especially when compared to the positive control and the Sigma standard. Fucoidan DES at a concentration of 50 μg/mL showed a greater quantity of invading cells, approximately 449 cells, corresponding to Figure 7A, revealing a strongly filled field, indicating great stimulation of migration. In contrast, the Sigma standard showed approximately 413 cells at the same concentration. This result can be attributed to the presence of small fractions of low molecular weight fucoidan, evidenced in Figure 4A, which exhibit greater interaction with cellular components, being associated with bioactive activities, such as the stimulation of migration [68].

However, fucoidan DES, even with low molecular weight percentages, presented lower purity percentages than Sigma. Therefore, this effect may not be linked only to the purity level of the material, but rather to the combination of the extract, such as sugar monomers, proteins, molecular weight and even residues of the eutectic solvent (LacAc:Gly), as evidenced by the FT‐IR spectrum (Figure 4B) [93, 101, 102]. For example, Jeong et al. evaluated the healing effects of hydrogels that were incorporated with fucoidan, which was isolated from the macroalgae *Ecklonia cava* [103]. The authors observed that hydrogels containing higher contents of crude fucoidan increased cell migration responses, in addition to demonstrating in vivo that this material presented better performance and complete acceleration in wound healing.

On the other hand, it can be observed that there was a reduction in the invasion capacity of fucoidan DES at a concentration of 75 μg/mL, approximately 397 cells that would invade, when compared to the concentration of 50 μg/mL. Although there was a decrease in the effect, it was not considered statistically significant compared with the positive control, presenting equally effective results in stimulating cell invasion. The result obtained can be reinforced by the viability of the bioactive (Figure 6A), where this range is suitable for cell migration [93, 104]. Therefore, the results shown in Figures 7A and B indicate that the fucoidan extracted by the eutectic solvent at both concentrations presented similar or superior performance to that of Sigma's fucoidan, which may indicate that extraction via DES preserved the biological properties of fucoidan. Furthermore, the concentration of fucoidan DES at 50 μg/mL surpassed the performance of fucoidan Sigma, proving the potential of the bioactive in cell regeneration and healing [105]. Corroborating the properties of fucoidan, the authors Wen et al. evaluated the healing effect of fucoidan and observed that the bioactive promotes angiogenesis and formation of granulation tissues, through signaling pathways such as AKT/ Nrf2/ HIF‐1*α* [105].

**FIGURE 8:**
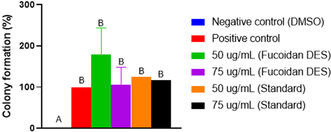
Evaluation of cellular clonogenic formation after treatment with fucoidan DES and the standard at different concentrations (50 and 75 µg/mL). Statistical data were considered significant at *p* < 0.05. Different capital letters indicate significant differences between the experimental groups.

Furthermore, the literature demonstrates that fucoidan can induce increased fibroblast proliferation, contributing to an improved response in the wound healing microenvironment [95]. However, in the proliferation assays shown in Figure 6B, fucoidan DES showed no significant effect. Therefore, to investigate whether fucoidan could increase the viability and, consequently, the proliferation of L929 fibroblasts, a clonogenic assay was performed (Figure 8). This assay is important for evaluating the impact of the extraction method on the biological properties of the polysaccharide.

The clonogenic assay results revealed that the negative control (DMSO) led to cell death after 10 days, which is expected given the high cytotoxicity of DMSO. However, fucoidan DES promoted a higher number of colonies compared to the positive control and Sigma's standard fucoidan. This increase suggests that fucoidan extracted with LacAc:Gly not only sustained cell viability but also increased the cells’ proliferative capacity over time [93, 99]. Because the colony formation assay assesses the ability of a single cell to proliferate into a colony, the results presented in Figure 7 indicate that fucoidan DES may provide more favorable conditions for cell renewal [106]. This property is essential for tissue regeneration, in which cell viability and proliferation play a key role in effective wound healing [107].

As shown, the 50 µg/mL concentration of fucoidan DES produced the best result, increasing cell survival by approximately 79% when compared to the positive control. This result was superior to that of Sigma's commercial fucoidan, which showed 25% and 17% increases at concentrations of 50 and 75 µg/mL, respectively. These results reinforce the effectiveness of the eutectic solvent extraction method, as the result surpasses that of Sigma's commercial standard. The observed increase in proliferation may be associated with a combination of factors, including the fact that the fucoidan extracted with eutectic solvent is less purified than Sigma's standard. Consequently, the extract may contain additional components, such as sugar monomers, proteins, and residual lactic acid and glycerol from the extraction process, which may collectively contribute to the observed biological effect [107]. In this context, it is possible that, at lower concentrations, the bioactive efficiently activates proliferation pathways, while at higher concentrations it may interfere with or saturate these processes, reducing their effectiveness [105, 108].

Therefore, the clonogenic assay resulting from fucoidan extracted by LacAc:Gly indicated an increase in colony formation at both concentrations (50 and 75 µg/mL), compared to Sigma's standard fucoidan, indicating a greater proliferation capacity over 10 days. However, in the proliferation assay (Figure 6B), which evaluated the cell growth property over 72 h, the results of fucoidan DES were not as evident, indicating that fucoidan DES did not show significant proliferation in the short term. However, as fucoidan DES showed a positive effect in the clonogenic assay, it indicates that the bioactive preserved viability and proliferation more effectively in the long term, with no immediate proliferation being noticeable [93, 99]. For example, the authors Ghahtan et al. studied the therapeutic potential of fucoidan in wound healing and found that fucoidan interacts with the TGF‐*β*1 protein and increases fibroblast growth rates in the wound, in addition to proposing maintenance of the structure of tissue cells [12, 107].

**FIGURE 9:**
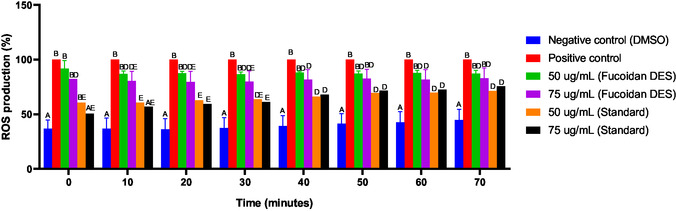
Reactive oxygen species production assay on treatments for fucoidan DES (50 and 75 µg/mL), standard fucoidan (50 and 75 µg/mL), negative control (DMSO) and positive control, over a period of 70 min. Statistical data were considered significant at *p* < 0.05. Different capital letters indicate significant differences between the experimental groups.

ROS are essential for wound healing. These include superoxide (O_2_^−^), hydroxyl radicals (•OH), hydroxide ions (OH^−^), hydrogen peroxide (H_2_O_2_), and peroxide (O_2_^2−^). In the wound healing process, ROS are involved in several steps, including antibacterial activity [109], angiogenesis, cell growth and migration, and ECM deposition [110, 111, 112]. However, ROS production requires tight regulation, as excessively high levels can lead to oxidative stress and tissue damage, while insufficient levels of ROS can impair cellular and molecular processes essential for physiological healing. In this context, maintaining balanced ROS levels increases antioxidant capacity and aids in wound healing [113]. Therefore, the aim of this analysis is based on investigating the role of fucoidan DES in modulating ROS production in L929 fibroblast cells (Figure 9).

The ROS results obtained, presented in Figure 9, showed that the cells treated with fucoidan extracted with the eutectic solvent (LacAc:Gly) presented high ROS production (approximately 91%) at the initial time, close to the positive control. However, the percentage tended to decrease over time, mainly at a concentration of 50 μg/mL, which was approximately 87%. This result indicates that fucoidan DES may not present a rapid antioxidant effect, but has the ability to reduce oxidative stress gradually. The molecule did not present high values in the DPPH, ABTS and FRAP antioxidant analyses at a concentration of 0.67 μg/mL, resulting in 30.4%, 25.8%, and 690 μg**/**μg, respectively (Table 3). Furthermore, it was observed that, in the results obtained in the proliferation and clonogenic assays (Figures 6B and 8), fucoidan DES did not present immediate responses; however, it was able to be viable for the cell to be able to form colonies in the long term. This response may indicate that the bioactive has an adaptive effect initially, and gradually enhances the cellular stimulus, favoring tissue regeneration [93, 99]. The authors Bittkau et al. [96] reported that the increase in ROS in cells treated with fucoidan may indicate the purity, concentration and also the incubation time of the biomolecule, as fucoidan DES had positive effects over time, as evaluated in the clonogenic assay. Furthermore, standard fucoidan presented lower percentages than fucoidan DES, with values close to those of the negative control, presenting moderate percentages.

Because Sigma's fucoidan presented higher purity than the one studied, it was able to present immediate properties, reducing oxidative stress [67, 89]. It is worth mentioning that moderate levels of ROS are important for the healing process and tissue regeneration [113]. Excess neutralization of cellular oxidative stress can slow down the healing process, while moderate neutralizations aid in the migration and proliferation of cells in wounds [113]. For example, the authors Wang et al. evaluated that in moderate concentrations of cellular oxidative stress, they affect the effectiveness of platelet activation at the wound site, accelerating healing [114]. Although fucoidan DES did not show immediate effects, it triggered inhibition over time, which may indicate that fucoidan extracted by LacAc:Gly has properties that can increase the activity of SOD and GSH enzymes, and reduce MDA markers, accelerating the proliferation and migration of cells in the wound [115, 116].

## 3 Conclusion

The extraction of fucoidan from *Sargassum muticum* using a deep acidic eutectic solvent based on lactic acid and glycerol proved to be a sustainable and efficient alternative to conventional extraction methods. The extraction of fucoidan from *Sargassum muticum* using a deep acidic eutectic solvent based on lactic acid and glycerol proved to be a sustainable and efficient alternative to conventional extraction methods. The green extraction process showed high selectivity for sulfated polysaccharides, combined with low toxicity and the potential application of the DES in sustainable formulations. The optimization step highlighted temperature as the most relevant factor, significantly influencing both the yield and purity of the extracted fucoidan. Although the predictive model indicated more severe conditions, the experimental results demonstrated superior performance at 60°C, with 4% water and a solid/liquid ratio of 0.117 *S*/*L.* Under these conditions, an extract with a high sulfate content and low hexuronic acid content was obtained, indicating greater selectivity and structural preservation of the biomolecule. Thus, process optimization resulted in obtaining a fucoidan with structural characteristics typical of brown algae, exhibiting heterogeneous molecular mass and compatible spectroscopic profiles, evidenced by FT‐IR bands and NMR chemical shifts, which indicated a high concentration of L‐fucose and *β*‐D‐galactose residues. Furthermore, the extracted fucoidan showed antioxidant potential, notably the high value observed in the FRAP assay, reaching approximately 690 μg/μg of inhibition. Regarding biological activity, the biomolecule demonstrated good cell viability at concentrations of 50 and 75 μg/mL. Although no immediate effect on cell proliferation was observed, as indicated by the proliferative analysis, the results of the clonogenic assay revealed greater colony formation at both concentrations evaluated when compared to Sigma's fucoidan. These findings indicate that fucoidan obtained through DES exhibits a more pronounced long‐term biological effect. Additionally, fucoidan extracted using DES, at a concentration of 50 μg/mL, showed high invasive capacity, comparable to its regenerative activity, promoting accelerated healing and cell growth. Despite the promising regenerative performance, further investigations are needed to deepen the purity of the material, including purification steps to reduce possible impurities such as uronic acids and possible residual toxic elements. Even so, the results obtained demonstrate that the extraction of fucoidan using acidic eutectic solvents constitutes an innovative, efficient, and sustainable strategy for the valorization of invasive macroalgae.

## 4 Materials and Methods

### 4.1 Biological Material

The macroalgae *S. muticum* was collected from the Caribbean in Mexico in January, during winter (20° 37′ 53″ N, 87° 04′ 23″ O). Initially, the samples were washed with distilled water to remove the salts and surface impurities. Subsequently, the biomass was treated with 92.3° ethanol (at a ration of 8.7 mL/g) and filtered using a 300 mesh nylon filter. The resulting precipitate was then dried in an oven at 55°C ± 2°C, ground to a mesh 35 and stored in a closed container at room temperature.

#### 4.1.1 Qualification and Elementary Analysis

The biomass *S. muticum* was subjected to physicochemical qualification analysis, where moisture content was determined bys the gravimetric method, proteins by the Kjeldahl method with nitrogen digester and distiller [117], ash content by the heating method at 555°C for 18 h in a muffle furnace [117], and lipids by the Soxhlet method, by evaporation of the solvent and measurement of residual material [117].

Additionally, a sequential extraction of fucoidan and alginate was performed. The dry biomass was treated with 0.5 M HCl at a ratio of 19.8 mL/g for 1 h at 60°C. After acid extraction, the supernatant was filtered and the fucoidan was precipitated by the addition of 80% (v/v) ethanol. The solid residue obtained after acid extraction was washed with distilled water and subsequently subjected to alkaline extraction with 0.1 M NaOH at the same ratio (19.8 mL g^−1^) for 2 h at 60°C. The alginate was recovered by adding 50% (v/v) ethanol to the alkaline supernatant. Both the fucoidan and alginate fractions were lyophilized [118, 119].

In the elemental analysis of *S. muticum* biomass, 3 mg of the sample was weighed on a high‐precision microbalance. The elements carbon, hydrogen, nitrogen, sulfur, and oxygen were quantified, the latter being estimated by the mass difference, using a Perkin‐Elmer CHNS/O 2400 Series II analyzer. The elements calcium, magnesium, iron, manganese, copper and zinc were determined by atomic absorption spectrophotometry. Nitrogen was quantified by the classical Kjeldahl method [117], while phosphorus, sulfur and boron were determined by colorimetry, and potassium (K) was determined by flame spectrophotometry.

### 4.2 Preparation of Deep Eutectic Solvents (DESs)

DESs were prepared by mixing two solvents, hydrogen donors (HBD) and hydrogen acceptors (HBA), using the molar ratios determined by COSMO‐RS, which can be seen in Table 1. The ratio of each of them was weighed and mixed in 2 mL Eppendorfs (Table 1), heated and stirred at 80°C at 900 rpm in a ThermoMixer for 2 h, until the entire solution was homogenized.

#### 4.2.1 COSMO‐RS Simulations

To use COSMO‐RS, the geometry and charge density of the individual molecules of a system must be optimized using DFT. In this work, each molecule was optimized using the COSMO‐BP‐TZVP template of the TmoleX 2024 (interface of TURBOMOLE), which includes a def‐TZVP basis set, DFT with the B‐P86 functional level of theory, and the COSMO solvation model (infinite permittivity). All COSMO‐RS calculations were performed using the software COSMOtherm 2024 with the BP_TZVP_24.ctd parametrization.

As COSMO‐RS is not suitable for calculations with ionic species, any salt applied as HBA was dealt with as ion pairs and then optimized using TmoleX. DESs were treated as binary mixtures of HBD and HBA at a fixed stoichiometric rate within the framework of COSMO‐RS. For any compound, its solubility in a solvent is inversely proportional to its activity coefficient in the system. As such, COSMO‐RS was used to predict the activity coefficients of monomers and oligomers from alginate and fucoidan in 24 DESs from 25°C to 85°C and infinite dilution.

#### 4.2.2 pH Analysis

The pH measurement was performed using a digital pH meter (Tecnal Tec‐5) after the preparation of the eutectic solvents, to assess whether they had an acidic or alkaline character, since fucoidan is extracted in more acidic environments.

#### 4.2.3 Thermogravimetric Analysis (TGA)

TGA was performed to evaluate the thermal decomposition of the eutectic solvents, identifying the appropriate temperature parameters to perform extractions without degrading the DES itself. This analysis was performed in an inert atmosphere, using the TGA‐50 Plus equipment (Shimadzu), with a nitrogen gas flow of 60 mL/min. The eutectic solvents were weighed to a mass of 6–8 mg in platinum crucibles. Thermal measurements were performed at 500°C at a heating rate of 10°C/min.

### 4.3 Fucoidan Extraction

Extractions were performed using a 1:10 (m/m) ratio, starting with 500 mg of algae and 5 mL of eutectic solvent, in duplicate, under conditions of agitation at 1000 rpm, 60°C for 2 h in a Thermomixer C. After extraction, the supernatant (containing the polysaccharides) was separated from the precipitate (algae) by filtration (Mesh 35), and the fucoidan was recovered by selective precipitation using 80% ethanol, remaining in contact for 12 h at 4°C. Then, a centrifugation was performed (10 min, 2000 rpm, 25°C), and the precipitate was lyophilized and analyzed. The remaining supernatant was refrigered for further analysis and testing.

#### 4.3.1 Chemical Characterization

The chemical characterization of fucoidan extracts was performed using spectrophotometric methods to determine total carbohydrates, uronic acids, sulfate groups, proteins, and phenolic compounds. In all assays, fucoidan samples were prepared at a concentration of 2 mg/mL. The total carbohydrate content was determined by the phenol‐sulfuric acid method [120]. Briefly, 200 µL of fucoidan solution was mixed with 200 µL of 5% (w/v) phenol, followed by the addition of 1 mL of concentrated sulfuric acid. The mixture was homogenized and allowed to react at room temperature for 30 min. Absorbance was measured at 490 nm. Fucose was used as a calibration standard. The uronic acid content was quantified using the carbazole method [121]. In this method, 200 µL of the sample was mixed with 1 mL of sulfuric acid containing sodium tetraborate and heated to 100°C for 10 min. After cooling, 40 µL of carbazole solution (0.1% w/v in ethanol) was added, followed by reheating for 10 min. Absorbance was measured at 525 nm. Glucuronic acid was used as a calibration standard. The sulfate content was determined by the barium chloride‐gelatin method after acid hydrolysis [122]. Fucoidan samples were hydrolyzed with hydrochloric acid and the hydrolysate was mixed with the barium chloride‐gelatin reagent. The reaction mixture was incubated at room temperature for 15 min and absorbance was measured at 360 nm. Potassium sulfate was used to construct the calibration curve. Protein content was determined by the Bradford assay [123]. In summary, 100 µL of the sample was mixed with 5 mL of Bradford reagent, incubated at room temperature for 10 min, and absorbance was measured at 595 nm. Bovine serum albumin (BSA) was used as a standard. Furthermore, total phenolic compounds were quantified by the Folin–Ciocalteu method [124]. For this assay, 200 µL of the sample was mixed with 1 mL of Folin–Ciocalteu reagent (1:10 with distilled water). After 5 min, 800 µL of sodium carbonate solution (7.5% w/v) was added. The mixture was incubated in the dark at room temperature for 30 min, and absorbance was measured at 750 nm. Gallic acid was used as a standard.

#### 4.3.2 Design Experimental (CCD)

To optimize the extractions performed with DESs, which resulted in higher yields, sulfate contents, total sugars, and low uronic acids, the Central Composite Design (CCD) was applied, a technique related to the response surface [125]. This method allows for the evaluation of the effects of independent variables on quantitative responses, determining linear influences and interactions.

In this study, three independent variables were selected: temperature (x1), solid‐liquid ratio (x2), and water (x3) (Table SA2). The experimental design comprised eight factorial points, six axial points and three repetitions at the central point, totaling 17 experiments (Table SA2). The coded levels of the variables were set at −1 and +1, where the axial points were (±*α*, 0, 0), (0, ±*α*, 0), and (0, 0, ±*α*). The value of *α* was 1.682, allowing rotation of the experimental design with three factors (Table SA2).

#### 4.3.3 Molecular Weight

The molecular weight of fucoidan samples was estimated by high‐performance gel permeation chromatography [126], in which the column was calibrated using appropriate heparin molecular weight markers and was used to characterize the molecular weight distribution of polydisperse samples or the peak molecular weight of monodisperse or nearly monodisperse heparin fractions. The same technology can be adapted for use with other glycans, such as fucoidan. Therefore, fucoidan samples (200 μg) were applied to analytical columns in series, TSK G4000 SWxl 7.8 mm × 30 cm and TSK G3000 SWxl 7.8 mm × 30 cm, connected to an HPLC equipment including the following components: isocratic HPLC pump; autosampler; valve injector, with appropriate loop size; RI detector; analog‐to‐digital data converter; computer with HPLC software.

#### 4.3.4 Physicochemical Characterization of Fucoidan by FT‐IR

FT‐IR spectroscopy analysis of polysaccharides (10 mg) was performed in an attenuated total reflection (ATR) window and analyzed in the wavelength range of 500–4000 cm^−1^ with 4 cm^−1^ resolution and averaging of 120 scans (Perkin Elmer Spectrophotometer) [127]. Vibration spectra were collected and analyzed using Essential FT‐IR 1.1.0.0 software (Madison, USA) and Sigma Plot software version 12.0 (Systat Software, Inc., California, USA).

#### 4.3.5 NMR Spectroscopy Analysis

The ^1^H and ^13^C NMR spectra of fucoidans were recorded using a Bruker DRX 500 MHz spectrometer with a triple resonance probe as described by De Araujo et al. [127]. Approximately 10 mg of the sample was dissolved in 1 mL of 99.6% deuterium oxide (D_2_O) as an external reference at 323 K with DO suppression by presaturation. The ^1^H NMR spectra were recorded using 16 scans. The spectrum HSQC (Edited Heteronuclear Single Quantum Correlation Spectroscopy) spectrum of ^1^H=^13^C (1024 × 256 points) was acquired with alternating rectangular phase pulses, globally optimized for decoupling. The chemical shifts of ^1^H and ^13^C were calibrated based on signals of trimethylsilyl propionic acid as a standard at 0 ppm. The spectra were processed using Top‐Spin 3.6.5 software (Bruker).

### 4.4 Antioxidant Activity

#### 4.4.1 DPPH Radical Scavenging Method

The antioxidant activity was determined by the DPPH free radical assay, which is responsible for evaluating the antioxidant capacity to reduce the purple radical to a colorless form [128]. Thus, a DPPH solution (0.024 mg/mL in methanol) was prepared, where 1800 µL was mixed with 45 µL of the sample. The reaction was carried out at all times in the dark for 30 min and the absorbance was read at a wavelength of 515 nm using an ELISA spectrophotometer, with consecutive readings, until the values were constant. Antioxidant capacity was determined as a percentage of DPPH inhibition, calculated by Equation (1).

(1) $$\text{Inhibition}\left(\%\right)=\left(\frac{ABS_{\text{control}}-ABS_{\text{sample}}}{ABS_{\text{control}}}\right)*100$$

#### 4.4.2 ABTS Radical Scavenging Method

The technique based on the ABTS method is based on the neutralization of the ABTS + cationic radical, characterized by the blue‐green coloration [129]. A 7 mM ABTS solution (192 mg in 50 mL of water) was prepared with 140 nM potassium persulfate solution (378.4 mg in 10 mL of water), which was kept in the absence of light. Afterward, 5 mL of the ABTS solution was combined with 88 µL of persulfate and left to stand in the dark for 16 h. After this interval, the solution was diluted in ethanol until it reached an absorbance range of 0.650 and 0.750, which was read at a wavelength of 734 nm. In the experiment, 15 µL of the sample or Trolox standard was added to 1500 µL of the ABTS solution and incubated for 6 min in the dark so that it could be read on the spectrophotometer. The antioxidant capacity was expressed in terms of the percentage inhibition of the ABTS radical, which was calculated using Equation (1) (DPPH).

#### 4.4.3 FRAP Method

The FRAP assay is capable of determining the antioxidant capacity through the conversion of Fe^3+^ ions into Fe^2+^, forming a blue complex [130]. For the analysis, a solution of 2 mM ferrous sulfate (27.8 mg/50 mL of water), 20 mM ferric chloride (5.4 g/L), and 0.3 M acetate buffer (pH 3.6) was prepared, formed by 3.1 g of sodium acetate and 16 mL of glacial acetic acid, which were diluted in 1 L of water. The FRAP reagent was obtained by mixing 10 mL of the buffer, 1 mL of 10 mM TPTZ and 1 mL of ferric chloride. In the assay, 15 µL of the sample was used and 285 µL of the reagent was added, and the samples were homogenized and incubated for approximately 30 min. The absorbance was read at 593 nm, with the reagent itself as the blank, and the results are expressed in µg gallic acid/µg dried algae. Calculated by Equation (2).

(2) $$C_{\text{Sample}}\left(\frac{\mathrm{\mu g}\;\text{gallic}\;\mathrm{acid}}{\mathrm{\mu g}}\right)=\frac{\text{Δ}ABS_{\text{Sample}}-b}{a}*\frac{\mathrm{\mu g}\;\text{gallic}\;\mathrm{acid}}{\mathrm{\mu g}\;\text{amostra}}$$

### 4.5 In Vitro Cellular Assays

#### 4.5.1 Cell Line and Culture

Normal L929 cells (fibroblasts) were maintained in RPMI medium supplemented with 10% (v/v) fetal bovine serum (FBS) and 1% antibiotics (glutamine and streptomycin) in an incubator with 5% CO_2_ at 37°C.

#### 4.5.2 Cell Viability

L929 cells (1 × 10^4^ cells/well) were seeded in 96‐well plates overnight in the presence of their culture media supplemented with 10% FBS. After this time, the cells were then treated with a fucoidan concentration curve (0.25; 0.50; 50; 75; 100; and 200 µg/mL) for 24 and 48 h in the presence of their culture medium without FBS, with the positive (no treatment) and negative (DMSO) standards. Cell viability was quantified in a Varioskan Lux microplate reader (LTC‐FLUO‐001) through the MTT assay, at a wavelength of 570 nm [131].

#### 4.5.3 Proliferation

L929 cells (1 × 10^4^ cells/well) were seeded in 96‐well plates in the presence of their culture medium supplemented with 10% FBS. After this time, the cells were then treated with a fucoidan concentration curve (50 and 75 µg/mL) for 24, 48, and 72 h in the presence of their culture medium with FBS, with the positive (no treatment) and negative (DMSO) standards. Proliferation was quantified in a Varioskan Lux microplate reader (LTC‐FLUO‐001) through the MTT assay, at a wavelength of 570 nm [131].

#### 4.5.4 Clonogenic Assay

L929 cells were seeded in 24‐well culture plates (4 × 10^4^ cells/cm^2^) in RPMI medium containing 10% FBS and were grown for 24 h. Then, the cells were treated with fucoidan (50 and 75 µg/mL) for 48 h. The cells were maintained in medium changes every 3 days throughout the assay, which lasted for 10 days. The wells were washed and the cells were fixed with 1.1% glutaraldehyde diluted in PBS for 10 min at room temperature. The wells were washed three times with PBS and incubated with crystal violet (0.1%) for 10 min, also at room temperature. The number of colonies was quantified by counting ten fields in each well, using an inverted microscope. Formations with fewer than eight cells were not considered colonies [132].

#### 4.5.5 Measurement of Reactive Oxygen Species

L929 cells (3 × 10^5^ cells/cm^2^) were seeded in black 96‐well plates for 2 h in RPMI medium supplemented with 10% FBS. After this period, the cells were washed with PBS to completely remove phenol red, as it can interfere with ROS measurement by generating fluorescence during probe metabolism. After this, the cells were treated for 1 h with the CM‐H2DCF DA probe (which, in general, ROS detection shows some selectivity for H_2_O_2_. Excitation and emission wavelengths were monitored at 495 and 525 nm, respectively. The probe was used at a final concentration of 5 µM. After this, the cells were washed with PBS to remove the probes and transferred to the Envision microplate reader to measure the fluorescence intensity emitted by ROS production. This measurement was made at different time intervals (0, 30, 60, and 70 min) [15].

#### 4.5.6 Cell Invasion Assay

In the cell invasion assay, 8 μm transparent porous inserts (Merck, cat# CLS3422‐48EA) in 24‐well plates were coated with 200 μg/mL of reduced growth factor Geltrex (Gibco, cat# A1413201), diluted in TBS buffer (10 mM Tris, pH 8.0, containing 0.7% NaCl), for 2 h at 37°C. Then, L929 cells (2.5 × 10^4^ cells/cm^2^) were seeded on the polymerized matrix in the presence of fucoidan extracted by LacAc:Gly or commercial fucoidan Sigma (both 50 and 75 μg/mL), diluted in culture medium. The lower compartment of the wells received culture medium supplemented with 5% FBS. After 18 h, the cells were removed from the top of the filter using cotton swabs and the inserts were fixed with 3.7% formaldehyde. Cells were stained with DAPI and counted using an EVOS‐fl fluorescence microscope at 20x magnification in 10 representative fields per condition [133].

### 4.6 Statistical Analysis

All experimental procedures were performed in triplicate and the results are expressed as the mean ± standard deviation. The means were compared by Analysis of Variance (ANOVA) followed by a one‐way test using GraphPad Prism software (version 8.0 Inc., California, USA). Statistical data were considered significant at *p* < 0.05, where different letters indicate differences between experimental groups.

## Author Contributions

Conceptualization: **Marina Leite** and **Bernardo Ribeiro**. Methodology: **Marina Leite**, **Francisca Silva**, **Helga Gomes**, **Thiago Cahú**, **Antônio Lima**, and **Afonso Cunha**. Validation: **Marina Leite** and **Bernardo D. Ribeiro**. Formal analysis: **Marina Leite**, **Francisca Silva**, and **Antônio Lima**. Investigation: **Marina Leite**. Resources: **Bernardo D. Ribeiro**, **Mauro S. G. Pavão**, **Thiago Cahú**, and **Paulo A. S. Mourão**. Preparation of the original draft: **Marina Leite**. Review and editing: **Marina Leite**, **Francisca Silva**, **Mauro S. G. Pavão**, and **Bernardo D. Ribeiro**. Supervision: **Bernardo D. Ribeiro** and **Mauro S. G. Pavão**. Obtaining funding: **Bernardo D. Ribeiro**. All authors read and agreed to the published version of the manuscript.

## Supporting Information

Additional supporting information can be found online in the Supporting Information section. **Supporting** **Fig. S1**: Representation of profiles for predicted values and desirability. **Supporting** **Fig. S2**: Pareto diagram of fucoidan optimization in terms of A) Yield, B) Hexuronic Acid, C) Hexose (total sugars) and D) Sulfate extracted by LacAc:Gly (1.20). **Supporting** **Table S1**: Screening of the affinity between deep eutectic solvents and fucoidan fractions via COSMO‐RS at 60°C. **Supporting** **Table S2**: Experimental factors with their respective coded levels. **Supporting** **Table S3**: Experimental design with the eutectic solvent LacAc:Gly (1.20). **Supporting** **Table S4**: Price for production of DESs per 10 mL. **Supporting** **Table S5**: Results obtained from the optimization of the DES LacAc:Gly (1.20). **Supporting** **Table S6**: Molecular weight of fucoidan extracted *S.* *muticum* using LacAc:Gly (1.20). **Supporting** **Table S7**: Structural FT‐IR absorption spectra of standard Fucoidan and Fucoidan DES. **Supporting** **Table S8**: ^13^C and ^1^H NMR data of fucoidan extracted of *Sargassum muticum.*

## Conflicts of Interest

The authors declare no conflicts of interest.

## Supporting information

Supplementary Material

## Data Availability

The data that support the findings of this study are available from the corresponding author upon reasonable request.
