# Supplementary material for: Extraction and Optimization of Fucoidan From Sargassum muticum Using Acidic Eutectic Solvents and Evaluation of Their Effects on Fibroblast Cell Lines
Source: ChemistryOpen. 2026 Apr 1;15(4):e202500448. doi: 10.1002/open.202500448 (PMC13045398; doi:10.1002/open.202500448)
Supplement: Supplementary file 1 — Supplementary Material [file OPEN-15-e202500448-s001.pdf]

Extraction and optimization of fucoidan from *Sargassum muticum* using acidic eutectic solvents and evaluation of their effects on fibroblast cell lines

Supplementary Material

Table A1: Screening of the affinity between deep eutectic solvents and fucoidan fractions via COSMO-RS at 60 °C.

LacAc:TMP	LacAc:Pro	LacAc:Gly	AcOH:TMP	AcOH:Gly	12PDO:PropAc	12PDO:LacAc	12PDO:AcOH
-0.09	0.09	-0.06	-0.28	-0.43	-0.02	-0.01	-0.12
0.45	1.87	0.21	0.17	0.21	-1.72	-0.28	-1.03
0.39	1.91	0.12	0.07	0.16	-2.14	-0.53	-1.36
0.09	1.92	-0.16	-0.28	-0.15	-3.14	-0.98	-2.09
0.23	1.64	0.19	-0.12	-0.34	-1.75	-0.07	-1.11
0.09	2.1	-0.19	-0.24	0.03	-3.08	-0.89	-2.07
0.04	2.04	-0.29	-0.33	-0.2	-3.53	-1.27	-2.39
0.76	3.42	0.43	0.09	-0.12	-3.7	-0.31	-2.39
0.03	3.02	-0.37	-0.66	-0.52	-5.83	-1.92	-3.98
0.28	2.3	0.37	-0.6	-0.73	-4.68	-1.11	-3.05
0.27	3.69	-0.44	-0.59	-0.57	-6.44	-2.16	-4.63
-0.64	2.55	-1.28	-1.53	-1.1	-8.4	-3.79	-6.04
0.67	4.34	-0.1	-0.21	-0.17	-5.93	-1.62	-4.26
-0.92	4.48	-1.83	-2.28	-1.8	-13.26	-5.33	-9.49
-0.12	0.12	0.06	-0.17	-0.36	-0.21	0.18	0.09
-3.35	-2.87	-3.28	-3.47	-2.32	-7.27	-5.48	-5.54
-3.95	-3.67	-3.79	-4.17	-2.89	-8.04	-6.2	-6.27
-9.0	-8.43	-8.84	-9.18	-6.71	-16.29	-13.05	-13.17
-11.25	-9.91	-11.52	-11.35	-8.69	-19.75	-16.07	-16.31
-19.49	-18.09	-19.6	-19.63	-15.23	-33.3	-27.3	-27.62
-11.95	-11.54	-11.52	-12.18	-8.52	-23.39	-18.19	-18.38
-33.63	-31.13	-34.09	-33.62	-26.1	-57.84	-47.26	-47.98
-35.23	-33.04	-35.36	-34.75	-26.32	-59.36	-48.85	-48.92

WILEY-VCH

15PDO:GlyAc	TMP:GlyAc	PropAc:13BDO	13BDO:LacAc	13BDO:GlycAc	Gly:GlycAc	12PDO:GlyAc	PropAc:TMP	PropAc:Gly
-0.01	-0.13	-0.27	-0.33	-0.13	-0.28	-0.44	0.03	-0.17
0.31	0.49	-3.95	-3.96	-0.56	-0.03	-1.31	-0.28	-1.18
0.16	0.5	-4.52	-4.52	-0.81	-0.13	-1.61	-0.52	-1.53
-0.13	0.1	-6.19	-6.17	-1.25	-0.36	-2.13	-1.04	-2.23
0.46	-0.06	-3.76	-3.74	-0.4	-0.14	-1.01	-0.21	-1.11
0.05	0.03	-6.25	-6.25	-1.08	-0.42	-2.2	-1.01	-2.18
-0.34	0.17	-6.98	-6.96	-1.54	-0.47	-2.49	-1.17	-2.53
0.97	0.16	-7.11	-7.0	-0.8	-0.4	-1.88	-0.57	-2.32
-0.37	-0.15	-10.55	-10.44	-2.35	-0.89	-3.57	-2.06	-4.11
-0.03	-0.26	-7.36	-7.18	-1.79	-0.3	-2.2	-1.69	-3.26
-0.17	0.3	-11.94	-11.87	-2.49	-1.2	-4.25	-1.96	-4.57
-1.74	-0.39	-13.91	-13.76	-4.2	-1.78	-5.61	-3.82	-6.34
0.44	0.65	-11.55	-11.51	-1.95	-1.01	-3.95	-1.42	-4.1
-2.04	-1.06	-21.83	-21.52	-6.08	-2.81	-8.17	-5.66	-9.67
0.08	-0.42	-0.64	-0.6	-0.08	0.05	0.01	-0.05	-0.11
-4.71	-2.64	-10.26	-10.16	-5.22	-2.3	-5.55	-5.77	-6.26
-5.37	-3.29	-10.63	-10.52	-5.88	-2.86	-6.19	-6.64	-7.07
-11.51	-7.7	-21.36	-21.18	-12.24	-6.92	-12.86	-13.68	-14.39
-14.05	-9.57	-26.63	-26.47	-15.06	-9.49	-16.28	-16.28	-17.51
-24.22	-16.79	-43.54	-43.18	-25.61	-15.99	-26.85	-27.88	-29.51
-15.92	-10.07	-30.77	-30.35	-16.95	-8.37	-17.18	-19.25	-20.17
-41.72	-28.85	-76.27	-75.58	-44.03	-27.54	-46.05	-47.89	-50.82
-43.55	-30.26	-78.61	-77.89	-45.35	-27.77	-47.19	-49.89	-52.13

WILEY-VCH

Components	PropAc:14BDO	LacAc:14BDO	AcOH:14BDO	14BDO:GlycAc	LacAc:15PDO	AcOH:15PDO	AcOH:13BDO
H_2O	0.26	0.22	0.0	-0.04	0.6	-0.11	0.24
Guluronic acid	-0.85	0.5	-0.38	-0.33	1.16	0.59	-0.57
Mannuronic acid	-1.22	0.34	-0.63	-0.53	0.98	0.49	-0.81
Diguluronic acid	-2.04	-0.08	-1.26	-1.07	0.86	0.11	-1.5
Dimannuronic acid	-0.8	0.5	-0.59	-0.43	1.58	0.22	-0.43
GM	-1.83	0.06	-1.09	-1.01	1.2	0.3	-1.39
MG	-2.37	-0.24	-1.48	-1.27	0.71	0.03	-1.83
Tetramannuronic acid	-2.02	0.72	-1.32	-1.01	2.43	0.71	-1.02
Tetraguluronic acid	-4.12	-0.59	-2.73	-2.23	0.94	-0.22	-2.85
MMGG	-3.57	-0.36	-2.49	-1.69	0.79	-0.54	-1.8
GGMM	-4.19	-0.45	-2.89	-2.41	1.56	0.0	-2.94
MGMG	-6.55	-2.08	-4.48	-3.73	-0.82	-1.23	-4.71
MGGM	-3.47	0.17	-2.34	-1.98	2.39	0.59	-2.33
GGMMGGMM	-10.12	-2.91	-7.04	-5.8	-0.43	-1.72	-7.17
fucose	-0.07	0.27	-0.04	0.03	0.52	-0.07	0.05
Fucose 2,3-sulfato	-7.11	-4.87	-5.11	-4.47	-4.99	-3.95	-5.98
Fucose 2,4-sulfato	-7.97	-5.66	-5.89	-5.17	-5.81	-4.72	-6.52
Fucoidan dimer α 1,3	-16.15	-12.19	-12.34	-11.19	-12.4	-10.22	-13.91
Fucoidan dimer α 1,3, 1,4	-19.14	-14.68	-14.93	-13.89	-14.66	-12.22	-17.11
Fucoidan tetramer 1,3	-32.89	-25.6	-25.84	-23.79	-26.01	-21.61	-29.23
Fucoidan tetramer 1,3, 1,4	-23.6	-17.28	-17.52	-15.4	-17.95	-14.24	-19.83
Fucoidan octamer 1,3	-57.04	-44.42	-44.7	-41.15	-45.05	-37.16	-50.99
Fucoidan octamer 1,3, 1,4	-58.98	-46.24	-45.82	-42.23	-47.24	-38.46	-53.19

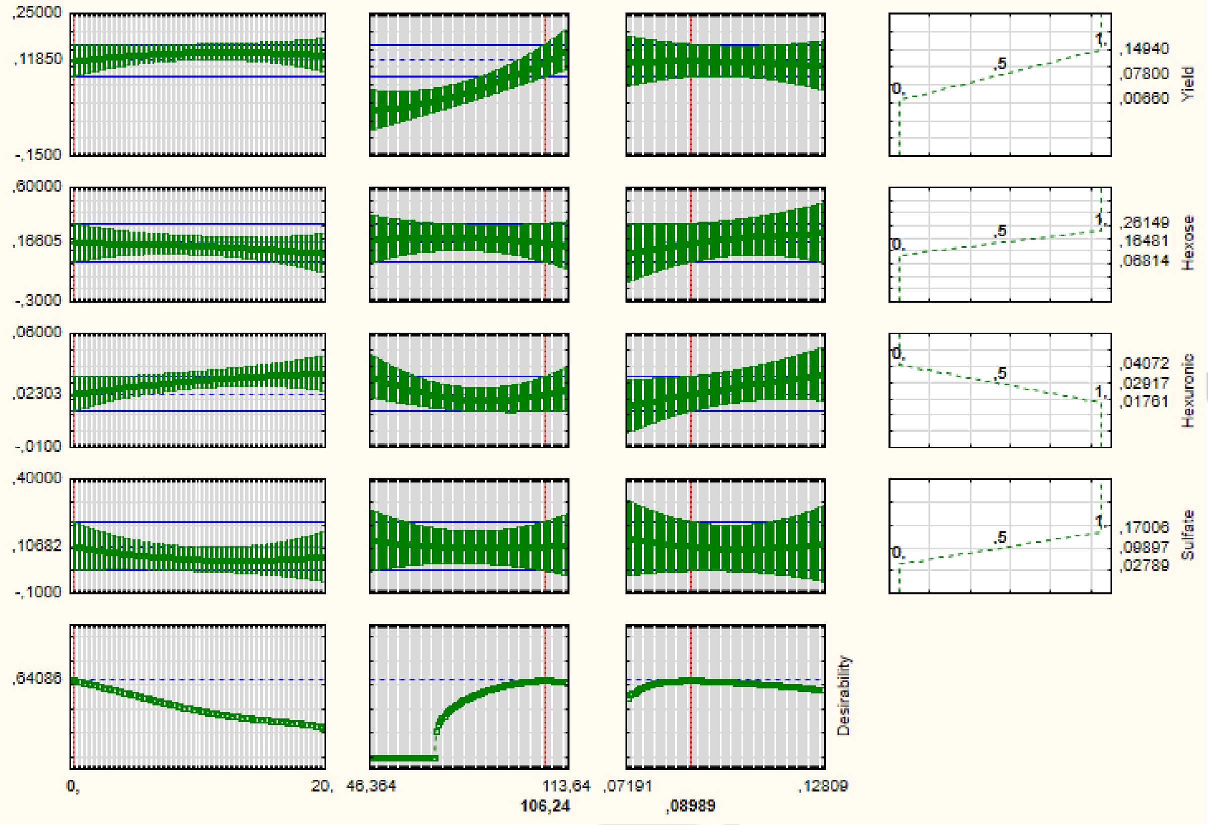

Figure A1: Representation of profiles for predicted values and desirability.

A)

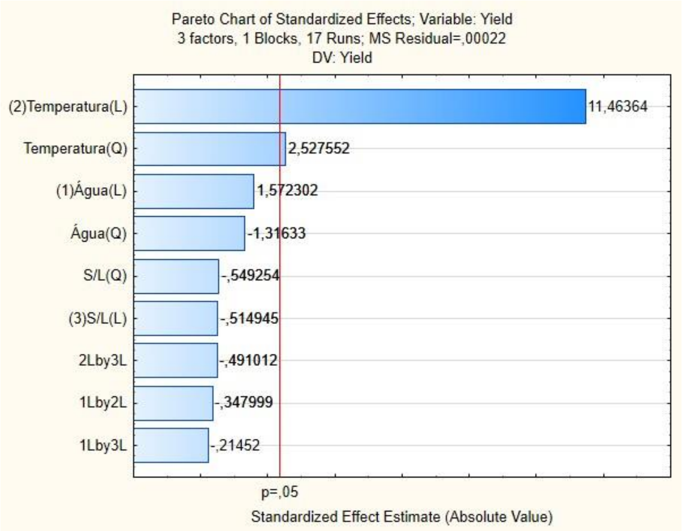

B)

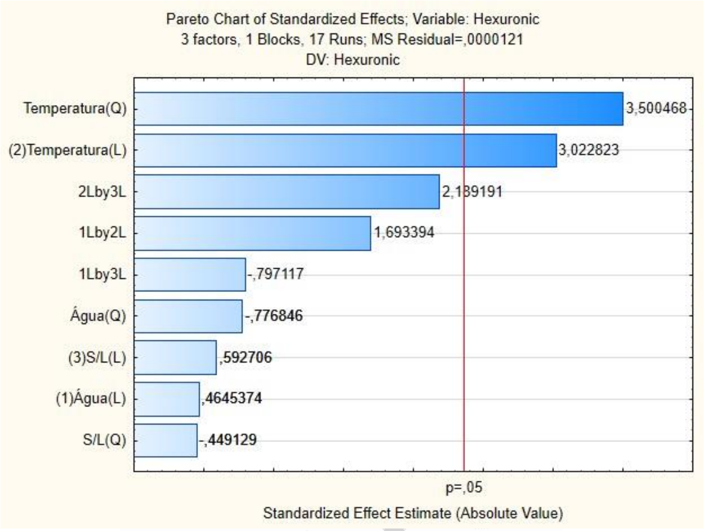

C)

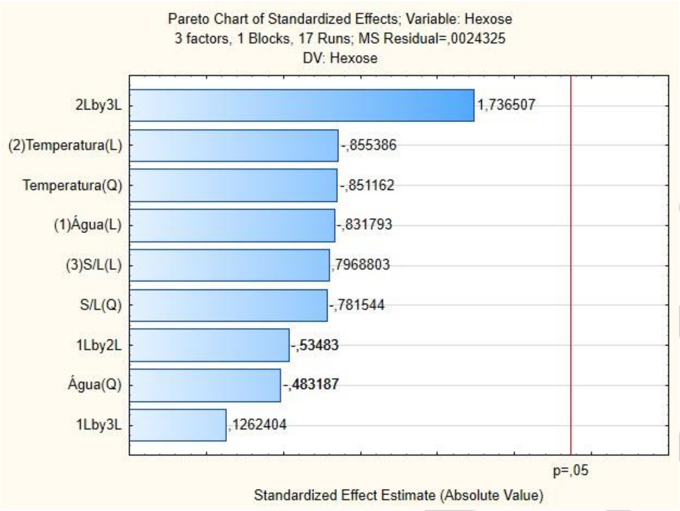

D)

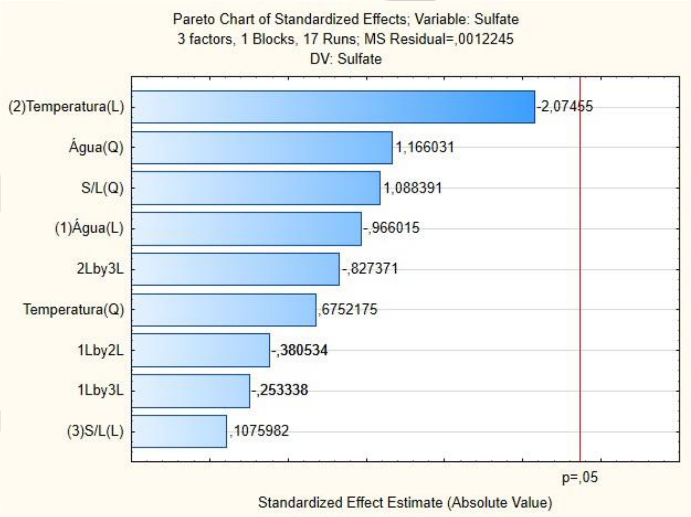

Figure A2: Pareto diagram of fucoidan optimization in terms of A) Yield, B) Hexuronic Acid, C) Hexose (total sugars) and D) Sulfate extracted by LacAc:Gly (1.20).

Table A2: Experimental factors with their respective coded levels.

Factors	Code	Variable levels				
		-α	*-1*	*0*	*1*	*+α*
Temperature (°C)	X1	46.36	60.00	80.00	100.00	113.63
Proportion (S/L)	X2	0.07	0.08	0.10	0.12	0.13
Water	X3	0.00	4.00	10.00	16.00	20.00

WILEY-VCH

Table A3: Experimental design with the eutectic solvent LacAc:Gly (1.20).

Experimental Order	Water (%)	Temperature (°C)	Proportion (S/L)
1	4	60	0.0833
2	4	60	0.1167
3	4	100	0.0833
4	4	100	0.1167
5	16	60	0.0833
6	16	60	0.1167
7	16	100	0.0833
8	16	100	0.1167
9	0	80	0.1000
10	20	80	0.1000
11	10	46	0.1000
12	10	114	0.1000
13	10	80	0.0719
14	10	80	0.1281
15	10	80	0.1000
16	10	80	0.1000
17	10	80	0.1000

WILEY-VCH

Table A4: Price for production of DESs per 10 mL.

Solvents	Molar Ratio	Price (10 mL)
12PDO : AcOH	1.20	$0.36
12PDO : LacAc	1.80	$0.33
12PDO : PropAc	1.40	$0.52
AcOH : Gly	1.70	$0.70
AcOH : TMP	2.10	$0.55
LacAc : Gly	1.20	$0.77
LacAc : Pro	1.90	$0.70
LacAc : TMP	1.50	$0.55
PropAc : Gly	1.80	$0.90
PropAc : TMP	2.10	$0.78
12PDO : GlycAc	3.10	$3.46
Gly : GlycAc	1.30	$7.61
13BDO : GlycAc	2.10	$5.63
13BDO : LacAc	1.40	$0.53
PropAc : 13BDO	1.10	$0.70
TMP : GlycAc	1.10	$7.47
15PDO : GlycAc	1.90	$5.90
AcOH : 13BDO	1.10	$0.52
AcOH : 15PDO	1.70	$0.95
LacAc : 15PDO	1.10	$1.12
14BDO : GlycAc	1.50	$5.65
AcOH : 14BDO	1.50	$0.34
LacAc : 14BDO	1.00	$0.29
PropAc : 14BDO	1.50	$0.55

WILEY-VCH

Table A5: Results obtained from the optimization of the DES LacAc:Gly (1.20).

Experimental Order	Water (%)	Temperature (°C)	Proportion (S/L)	Yield (mg/mg)	Hexose (mg/mg)	Uronic acid (mg/mg)	Sulfate (mg/mg)
1	4	60	0.0833	0.098	0.1681	0.023	0.099
2	4	60	0.1167	0.066	0.1758	0.023	0.170
3	4	100	0.0833	0.118	0.1481	0.021	0.090
4	4	100	0.1167	0.1168	0.1802	0.029	0.062
5	16	60	0.0833	0.0166	0.2221	0.024	0.114
6	16	60	0.1167	0.0212	0.1420	0.018	0.115
7	16	100	0.0833	0.1298	0.0681	0.028	0.028
8	16	100	0.1167	0.1118	0.2058	0.035	0.046
9	0	80	0.1000	0.0228	0.2615	0.025	0.077
10	20	80	0.1000	0.0574	0.1913	0.023	0.074
11	10	46	0.1000	0.0268	0.2255	0.033	0.060
12	10	114	0.1000	0.1494	0.1956	0.041	0.063
13	10	80	0.0719	0.0528	0.1992	0.025	0.088
14	10	80	0.1281	0.0466	0.2277	0.025	0.059
15	10	80	0.1000	0.0628	0.2105	0.022	0.067
16	10	80	0.1000	0.049	0.2471	0.026	0.045
17	10	80	0.1000	0.0716	0.1791	0.023	0.049

WILEY-VCH

Table A6: Molecular weight of fucoidan extracted *S. muticum* using LacAc:Gly (1.20).

Retention time (min)	MW (Da)	MW (KDa)	MW (Log)
38.88	104722.47	104.72	5.02
34.12	168465.22	168.47	5.23

WILEY-VCH

Table A7: Structural FT-IR absorption spectra of standard Fucoidan and Fucoidan DES.

Structural building blocks	Infrared band (cm^{-1})	Reference
O-H group of monosaccharides	3400	[134]
C-H stretches	2900	[134]
C=O group of carboxylic acids	1640	[134]
COOH groups of hexuronic acids	1420	[135]
Asymmetric stretching S=O	1220-1240	[134-136]
C-O-C Groups	1030	[134]
C-O-S Groups	840	[136]
Constituent units of alginate	690	[136]

WILEY-VCH

Table A8: ^{13}C and ^{1}H NMR data of fucoidan extracted of *Sargassum muticum.*

Residue	Sugar linkage	Chemical shifts (ppm)[a]					
		H-1<br>C-1	H-2<br>C-2	H-3<br>C-3	H-4<br>C-4	H-5<br>C-5	H-6<br>C-6
A	→3.4)-α-L-Fuc (1→	5.41<br>98.7	3.8<br>71.2	4.11<br>73.9	4.47<br>81.2	4.46<br>66.6	1.37<br>19.65
B	L-Fucp (H2–H5)	5.23<br>99.9	4.59<br>78.6	4.15<br>68.3	3.81<br>84.0	-	-
C	→6)-β-D-Gal- (1→	4.48<br>102.9	3.78<br>71.7	3.86<br>77.8	4.47<br>81.7	3.85<br>77.8	3.92<br>62.3

[a]The data were btained from a combination of TOCSY ($^{1}H/^{1}H$) and HSQC ($^{1}H/^{13}C$) spectra at 500 MHz. The chemical shifts are based on Tetramethylsilane - TMS.

Reference

[134] Soto-Vásquez, M. R.; Alvarado-García, P. A. A.; Youssef, F. S.; Ashour, M. L.; Bogari, H. A.; Elhady, S. S. FTIR Characterization of Sulfated Polysaccharides Obtained from Macrocystis Integrifolia Algae and Verification of Their Antiangiogenic and Immunomodulatory Potency In Vitro and In Vivo. *Mar Drugs* **2023**, *21* (1). https://doi.org/10.3390/md21010036.

[135] Gieroba, B.; Kalisz, G.; Krysa, M.; Khalavka, M.; Przekora, A. Application of Vibrational Spectroscopic Techniques in the Study of the Natural Polysaccharides and Their Cross-Linking Process. *International Journal of Molecular Sciences*. 2023. https://doi.org/10.3390/ijms24032630.

[136] Vandanjon, L.; Burlot, A. S.; Zamanileha, E. F.; Douzenel, P.; Ravelonandro, P. H.; Bourgougnon, N.; Bedoux, G. The Use of FTIR Spectroscopy as a Tool for the Seasonal Variation Analysis and for the Quality Control of Polysaccharides from Seaweeds. *Mar Drugs* **2023**, *21* (9). https://doi.org/10.3390/md21090482.

WILEY - VCH
